# *Stenotrophomonas* comparative genomics reveals genes and functions that differentiate beneficial and pathogenic bacteria

**DOI:** 10.1186/1471-2164-15-482

**Published:** 2014-06-18

**Authors:** Peyman Alavi, Margaret R Starcher, Gerhard G Thallinger, Christin Zachow, Henry Müller, Gabriele Berg

**Affiliations:** Graz University of Technology; Environmental Biotechnology, Petersgasse 12, 8010 Graz, Austria; Institute for Genomics and Bioinformatics, Graz University of Technology, Graz, Austria

**Keywords:** Human pathogens, Stress Protecting Agent (SPA), Genomics, Transcriptomics

## Abstract

**Background:**

In recent years, the number of human infections caused by opportunistic pathogens has increased dramatically. Plant rhizospheres are one of the most typical natural reservoirs for these pathogens but they also represent a great source for beneficial microbes with potential for biotechnological applications. However, understanding the natural variation and possible differences between pathogens and beneficials is the main challenge in furthering these possibilities. The genus *Stenotrophomonas* contains representatives found to be associated with human and plant host.

**Results:**

We used comparative genomics as well as transcriptomic and physiological approaches to detect significant borders between the *Stenotrophomonas* strains: the multi-drug resistant pathogenic *S. maltophilia* and the plant-associated strains *S. maltophilia* R551-3 and *S. rhizophila* DSM14405^T^ (both are biocontrol agents). We found an overall high degree of sequence similarity between the genomes of all three strains. Despite the notable similarity in potential factors responsible for host invasion and antibiotic resistance, other factors including several crucial virulence factors and heat shock proteins were absent in the plant-associated DSM14405^T^. Instead, *S. rhizophila* DSM14405^T^ possessed unique genes for the synthesis and transport of the plant-protective spermidine, plant cell-wall degrading enzymes, and high salinity tolerance. Moreover, the presence or absence of bacterial growth at 37°C was identified as a very simple method in differentiating between pathogenic and non-pathogenic isolates. DSM14405^T^ is not able to grow at this human-relevant temperature, most likely in great part due to the absence of heat shock genes and perhaps also because of the up-regulation at increased temperatures of several genes involved in a suicide mechanism.

**Conclusions:**

While this study is important for understanding the mechanisms behind the emerging pattern of infectious diseases, it is, to our knowledge, the first of its kind to assess the risk of beneficial strains for biotechnological applications. We identified certain traits typical of pathogens such as growth at the human body temperature together with the production of heat shock proteins as opposed to a temperature-regulated suicide system that is harnessed by beneficials.

**Electronic supplementary material:**

The online version of this article (doi:10.1186/1471-2164-15-482) contains supplementary material, which is available to authorized users.

## Background

During the last years, the number of human infections caused by opportunistic pathogens has increased dramatically. One natural reservoir of opportunistic pathogens is the rhizosphere, the zone around roots that is influenced by the plant [1, 2]. Due to a high content of nutrients, this habitat is a ‘microbial hot-spot’, where bacterial abundances including those with strong antagonistic traits are enhanced [3]. Various bacterial genera, including *Burkholderia, Enterobacter, Herbaspirillum, Ochrobactrum, Pseudomonas, Ralstonia, Staphylococcus* and *Stenotrophomonas*, contain root-associated strains that can encounter bivalent interactions with both plant and human hosts [2]. Mechanisms responsible for colonization of the rhizosphere and antagonistic activity against plant pathogens are similar to those responsible for colonization of human organs and tissues, and pathogenicity [2]. Multiple resistances against antibiotics are not only found with clinical strains but also with strains isolated from the rhizosphere [4]. High competition, the occurrence of diverse antibiotics and secondary antimicrobial plant metabolites, and enhanced horizontal gene transfer and mutation rates in this microenvironment contribute to the high levels of natural resistances [5]. On the other hand, rhizosphere inhabitants have an enormous potential as biocontrol or stress protecting agents or as fertilizers for sustainable agriculture [6, 7].

*Stenotrophomonas maltophilia* is an emerging global pathogen and already one of the most common opportunistic pathogens in hospitals [8, 9]. A recent study shows that approx. 5% of the Gram-negative infections were caused by *S. maltophilia* in intensive care units in the United States. The two most common diseases caused by *S. maltophilia* are bacteremia and pneumonia, which are often associated with high mortality rates. *S. maltophilia* strains are characterized by multi-resistance to many antibiotics [10]. For a long time it was not possible to differentiate between the clinical and environmental *S. maltophilia* strains [4]. Using a polyphasic approach, Wolf *et al.* [11] were able to describe a new plant-associated species within the *S. maltophilia* complex. Interestingly, no human-pathogenic potential has ever been observed in this phylogenetically and ecologically closely related species [11]. Moreover, both species can be easily distinguished with regard to the production of the osmoprotective substance glucosylglycerol (only present in *S. rhizophila*) and the occurrence of specific multidrug-efflux pumps (only present in *S. maltophilia*) [12]. *S. rhizophila* is both rhizosphere- and phylloplane- competent and shows pronounced salt tolerance, and is hence a model bacterium among the plant growth-promoting rhizobacteria (PGPR) [13]. Plant growth promotion by *S. rhizophila* strain DSM14405^T^ (syn. strain e-p10) was observed under greenhouse conditions [14] and in the highly salinated soils of Uzbekistan at levels up to 180% [15]. However, *S. maltophilia* was a typical rhizosphere bacterium used as an efficient biocontrol agent, and until the 1980s, no capacity to cause infection had ever been reported. Now, the theory is established that the ancestors of virulent bacteria including *Stenotrophomonas*, as well as the origin of virulence and resistance determinants, lay most likely in the environmental microbiota [5]. It is now one of the main challenges to predict any risk for human health [16]. Currently, these potential risk factors are a main obstacle in registration procedures, especially in the European Union [17]. Next generation sequencing and the corresponding bioinformatic analyses have an enormous impact on our understanding of microbial communities and the host-microbe interactions [18, 19]. However, is it possible to use these techniques to solve this problem?

The objective of our study was to find out if there is a borderline based on the distinguishing features between the beneficials and pathogens within the genus *Stenotrophomonas*. Moreover, using genomics, transcriptomics and physiological assays, we try to predict the potential risk of the stress protecting agent *S. rhizophila* strain DSM14405^T^ for humans by studying its genetic potentials and comparing these with two *Stenotrophomonas* model strains, the human-pathogenic *S. maltophilia* K279a [20] and the plant-associated *S. maltophilia* R551-3 [21].

## Results

### Comparisons of plant and human-associated *Stenotrophomonas*genomes

General genomic features of *S. rhizophila* DSM14405^T^ were compared to the plant-associated *S. maltophilia* R551-3 and the human pathogenic *S. maltophilia* K279a (Table 1). The genome of *S. rhizophila* DSM14405^T^ consists of 4,648,976 nucleotides with a GC content of 67.26%, and has been predicted to code for 4,033 CDSs. Compared with *S. maltophilia* R551-3 and *S. maltophilia* K279a with each 4,573,969 n (4,039 CDSs) and 4,851,126 n (4,386 CDSs), respectively, the size of the genome and the predicted number of CDSs are slightly smaller. There is no plasmid present in *S. rhizophila* DSM14405^T^.

**Table 1:** **General genomic characteristics of** ***S. rhizophila*** **DSM14405** ^**T**^ **,** ***S. maltophilia*** **R551-3 and** ***S. maltophilia*** **K279a**

	S. rhizophila DSM 14405 ^T^*	S. maltophilia R551-3**	S. maltophilia K279a**
Number of bases	4,648,976 bp	4,573,696 bp	4,851,126 bp
G = C content (%)	67.26	66.3	66.3
Number of CDSs	4,033	4,039	4,386
Coding Percentage	88.5	89.4	88.8
Average of ORF Length	1,020 bp	1,013 bp	983 bp
rRNA	12(genes)	12(genes)	12(genes)
tRNA	72	73	74

*Information presented here corresponds to the original annotation. Alterations could occur due to possible updates.

**According to the genome information provided in the corresponding NCBI gbk-data.

**Figure 1:**
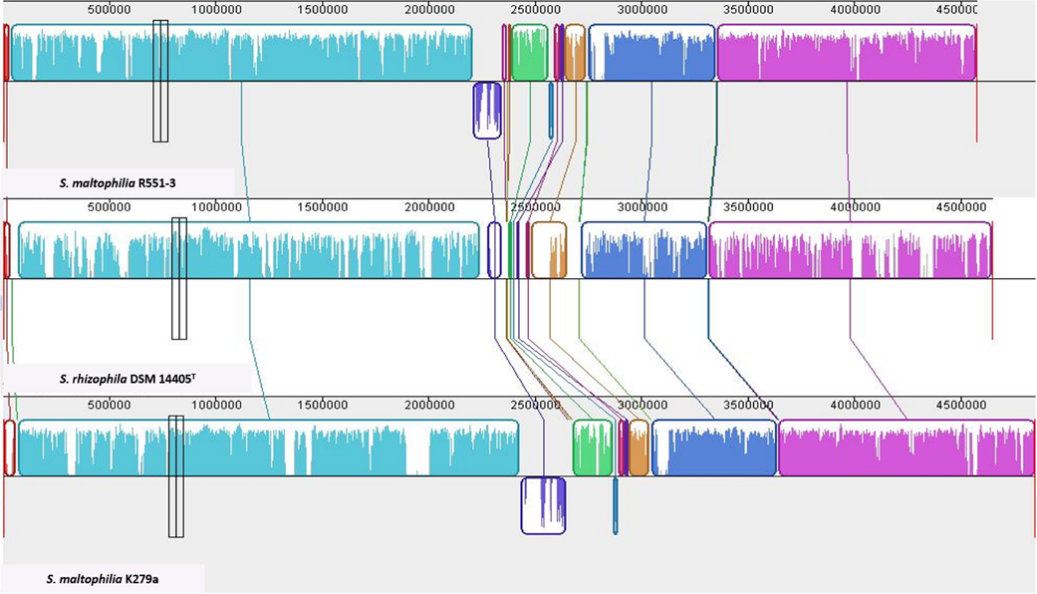
**Genome-scale comparison for the three** ***Stenotrophomonas*** **strains.** The plant-beneficial strains *S. maltophilia* R551-3 (top), *S. rhizophila* DSM14405^T^ (middle), and the human pathogenic *S. maltophilia* K279a (bottom). The original genomic sequence of *S. rhizophila* DSM14405^T^ was converted into its reverse complement to achieve the same direction for all three genomes. Homologous DNA segments among the strains are marked by boxes with the same color, while gaps correspond to non-homologous regions. There are vast regions of homology between the genome of *S. rhizophila* DSM14405^T^ and both *S. maltophilia* R551-3 and K279a. The figure was generated using nucleotide sequences of the genomes using Mauve 2.3.

**Figure 2:**
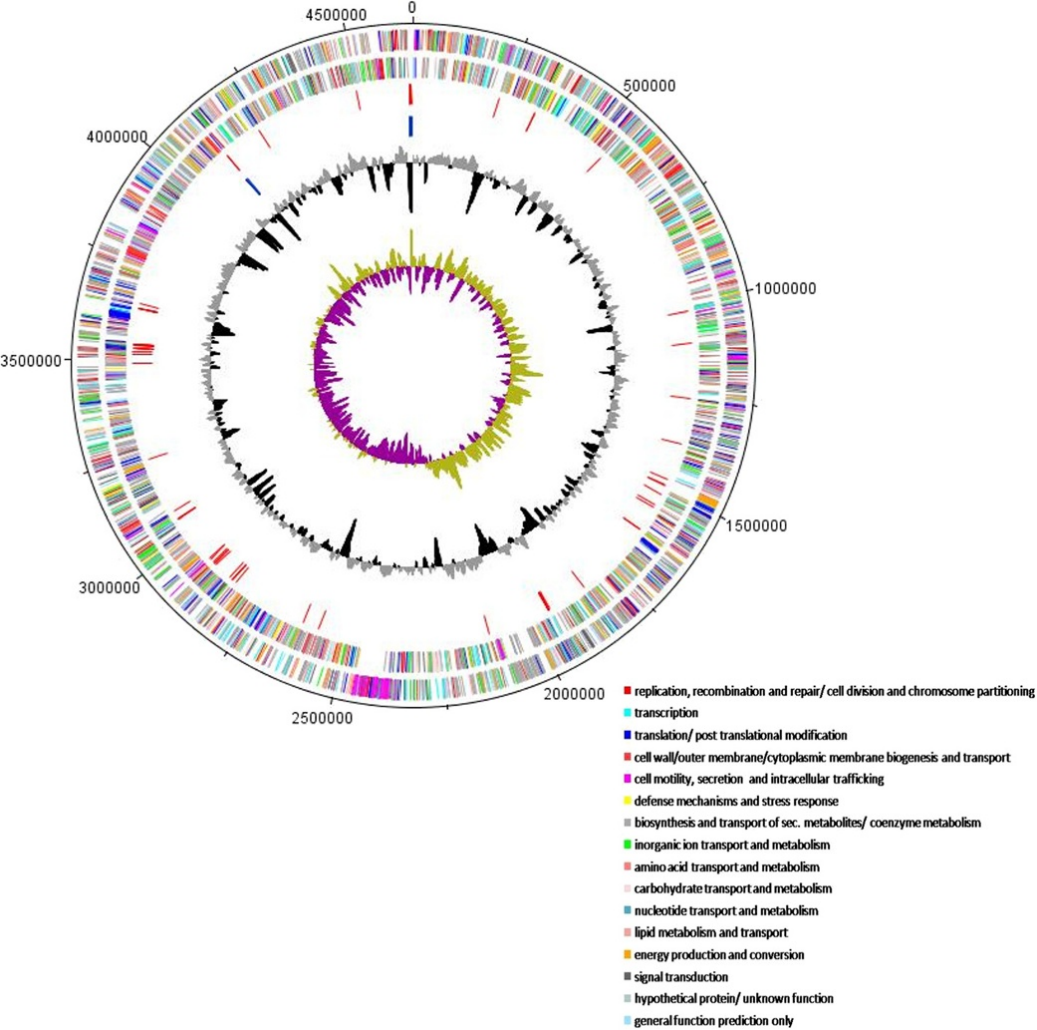
**Circular genome map of** ***S. rhizophila*** **DSM14405** ^**T**^ **.** Predicted coding sequences (CDSs) are assigned various colors with respect to cellular functions. The circles show from the outermost to the innermost: 1. DNA coordinates; 2, 3. Function-based color coded mapping of the CDSs predicted on the forward and reverse strands. Various functions are assigned different colors. 4. tRNA genes; 5. rRNA genes; 6. GC plot with regions above and below average in gray and black, respectively; 7. GC skew showing regions above and below average in dark yellow and magenta, respectively (window size: 10,000 bp). The circular genome map was constructed using DNAPlotter.

**Figure 3:**
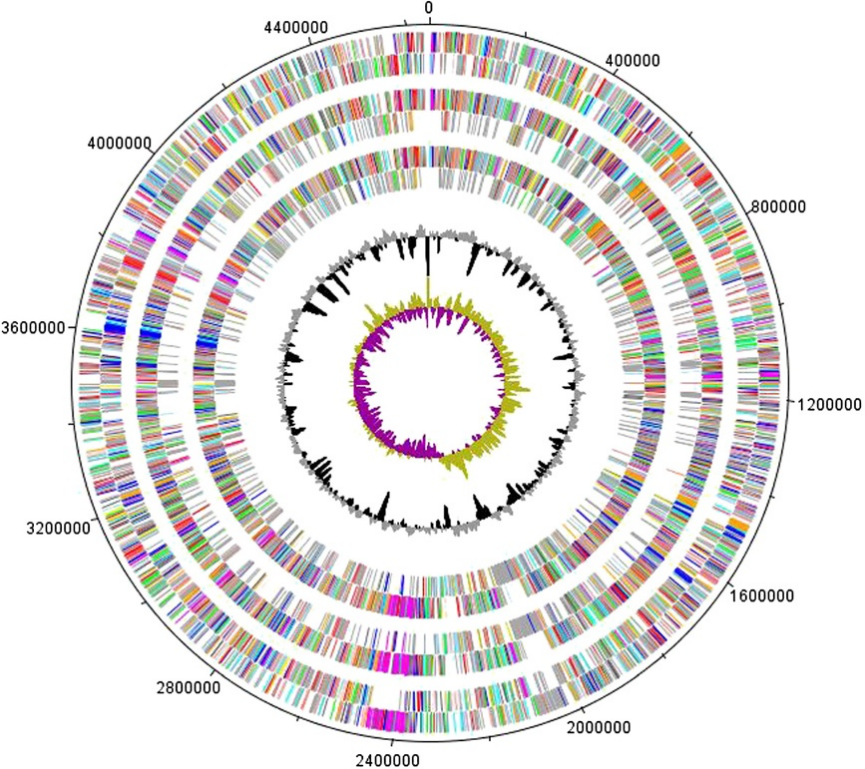
**Gene orthology analyses between** ***S. rhizophila*** **DSM14405** ^**T**^ **,** ***S. maltophilia*** **R551-3 and the clinical** ***S. maltophilia*** **K279a.** Circles show from the outermost to the innermost: 1. DNA coordinates; 2, 3. Function-based color-coded mapping of the CDSs predicted on the forward and reverse strands of the *S. rhizophila* DSM14405^T^ genome, respectively. 4. Orthologous CDSs shared between *S. rhizophila* DSM14405^T^ and *S. maltophilia* R551-3. 5. *S. rhizophila-*specific CDSs, compared with *S. maltophilia* R551-3. 6. Orthologous CDSs shared between *S. rhizophila* and *S. maltophilia* K279a. 7. *S. rhizophila-*specific CDSs, compared with *S. maltophilia* K279a. 8. GC plot depicting regions above and below average in gray and black, respectively; 9. GC skew showing regions above and below average in yellow green and magenta, respectively (window size: 10,000 bp). The assessment of orthologous CDSs was carried out using the reciprocal best BLASTp hit approach with an identity threshold of 30% and evalue of 10^-6^.

**Figure 4:**
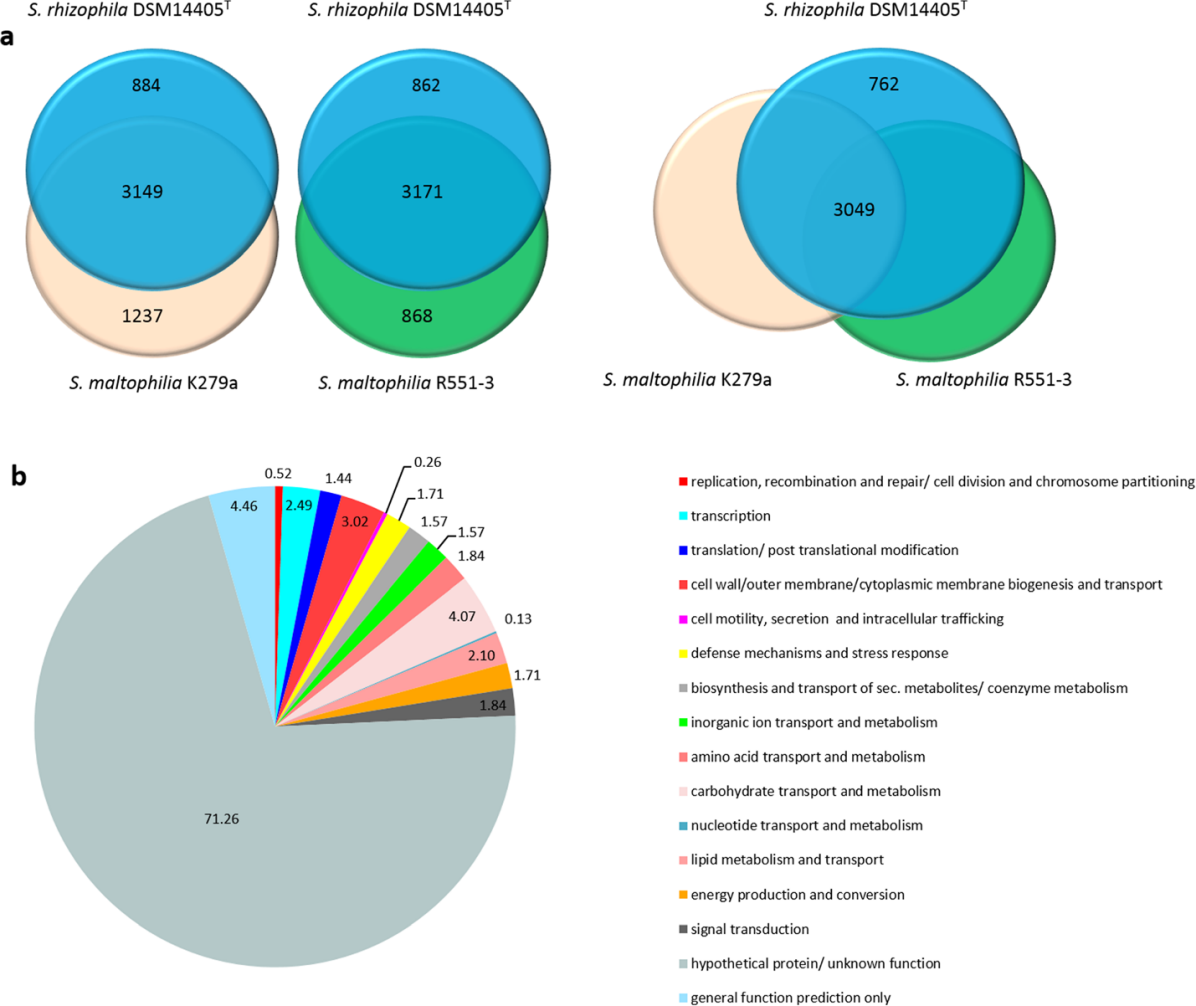
**Orthology analysis and the distribution of the** ***S. rhizophila*** **-specific CDSs with regard to cellular functions. a**: Venn diagram showing the number of CDSs shared between the three strains. *S. rhizophila* DSM14405^T^ was used as the reference genome. 3171 and 3149 CDSs are shared between *S. rhizophila* and *S. maltophilia* R551-3 and K279a, respectively. 3049 CDSs are shared between all three strains, as a trio-analysis of the three genomes revealed. 762 CDSs are absent in both *S. maltophilia* R551-3 and *S. maltophilia* K279a, and hence unique to *S. rhizophila*. **b**: Diagram showing the percentage distribution of the 762 *S. rhizophila*-specific CDSs with regard to the predicted cellular functions. Most of these are hypothetical genes (71.26%) or CDSs of general function (4.46%). Other *S. rhizophila*-unique genes showing a relative abundance are involved in carbohydrate transport and metabolism (4.07%), and cell wall, outer membrane, and cytoplasmic membrane biogenesis (3.02%).

Figure 1 shows genomic comparisons between the genome of *S. rhizophila* DSM14405^T^, *S. maltophilia* R551-3 [21] and *S. maltophilia* K279a [20]. Overall, there is a very high degree of sequence similarity between the genome of the plant growth-promoting *S. rhizophila* and both *S. maltophilia* R551-3 and the human-pathogenic K279a. The homology boxes are however separated by non-homologous regions. The whole genome sequence comparison between the three genomes was also performed using the Artemis Comparison Tool (ACT) which revealed the same result as in Figure 1 and is presented in Additional file 1: Figure S1. Figure 2 represents circular genomic map of *S. rhizophila* DSM14405^T^, wherein DNA coordinates are presented and coding sequences (CDSs) on both the leading and lagging strands are assigned colors with respect to functional gene groups. In addition, the figure depicts the position of tRNA and rRNA genes, and other characteristics of the *S. rhizophila* DSM14405^T^ genome such as GC content as well as the excess of C over G throughout the whole genome (GC skew) with the regions above and below average shown in different colors. Furthermore, gene orthology analyses were performed, in which *S. rhizophila* genes were compared with those of *S. maltophilia* R551-3 and K279a to detect orthologous genes shared among strains (Figure 3). In addition to orthologous genes, Figure 3 depicts the position and color-coded function of the *S. rhizophila* DSM14405^T^-specific genes. The numbers of orthologous and strain-specific unique genes are shown in the Venn diagrams (Figure 4a). As presented in the figure, the number of CDSs shared between *S. rhizophila* and *S. maltophilia* R551-3 or *S. maltophilia* K279a is 3171 (862 specific to *S. rhizophila*) and 3149 (884 specific to *S. rhizophila*), respectively. Moreover, the orthology analysis including all three strains revealed that 762 CDSs are unique to *S. rhizophila* DSM14405^T^, as these are absent from both *S. maltophilia* R551-3 and *S. maltophilia* K279a (Figure 4a). The percentage distribution of the 762 *S. rhizophila*-unique CDSs with regard to functional gene groups revealed that genes involved in carbohydrate transport and metabolism (4.07%), and the biogenesis and transport of cell wall, outer membrane or cytoplasmic membrane (3.02%) are of relatively high abundance (Figure 4b).

### Specific gene characteristics of *S. rhizophila*DSM14405^T^within the *Stenotrophomonas/Xanthomonas*group

#### Quorum sensing

Similar to various xanthomonads, *S. rhizophila* DSM14405^T^ does not possess a homoserine lactone-based quorum sensing system, but instead uses the *rpf*/DSF system for quorum sensing and cell-cell communication. The *rpf* (regulation of pathogenicity factors) gene cluster is responsible for the synthesis and perception of the DSF molecule which is a quorum sensing regulatory molecule of fatty acid nature with similarity to enoyl-CoA hydratase, and was first detected in *Xanthomonas* [22, 23]. The *rpfF* gene product, known as DSF synthase, is essential for the synthesis of DSF [24, 25]. Other members of the *rpf* gene locus (*rpfC*, *rpfG* and *rpfB*) have been revealed to each fulfill a particular function, with the RpfC/RpfG two-component system consisting of a sensory (RpfC) and regulatory (RpfG) component that are responsible for DSF perception and signal transduction, respectively [22, 26].

Similar to *Stenotrophomonas* strains K279a and R551-3, the core of the *rpf* gene locus of *S. rhizophila* DSM14405^T^ consists of four genes: *rpfB*, *rpfF*, *rpfC* and *rpfG*. Fouhy et al. [27] described the positions of these in the human-pathogenic *S. maltophilia* K279a, which we found to be similar to those in the plant-associated *S. maltophilia* R551-3. Moreover, in both K279a and R551-3, *rpfB* and *rpfF* are located on the lagging strand while *rpfC* and *rpfG* are located on the leading strand. In *S. rhizophila* DSM14405^T^, however, the *rpfB* and *rpfF* genes are located on the leading strand while *rpfC* and *rpfG* are on the lagging strand. In addition, in the genome of *S. rhizophila*, there is a 228-nucleotide gene of unknown function on the lagging strand between *rpfF* and *rpfB*, which extends from 2469447 to 2469674 and was annotated as Sr14405 DX03_10710. In addition, Sr14405 DX03_10710 is transcribed in the cell, as we detected the corresponding mRNA in a whole genome expression analysis approach (data not shown). There is no homologue to Sr14405 DX03_10710 in either *S. maltophilia* R551-3 or K279a, and its function in *S. rhizophila* DSM14405^T^ remains to be elucidated.

#### Flagella, fimbriae

Flagella and fimbriae-driven motility is crucial for biofilm formation and host-plant colonization by bacteria [28–30]. *S. rhizophila* DSM14405^T^ possesses several genes responsible for motility. A gene block encoding a flagellar apparatus was detected that includes 22 genes, and extends over the genome from Sr14405 DX03_10335 to DX03_10430 with most genes located on the leading strand. Another flagella-encoding gene block was also detected which includes 26 genes and is located between Sr14405 DX03_10470 and DX03_10585. Furthermore, two putative fimbriae gene clusters, Sr14405 DX03_04025-DX03_04040 and Sr14405 DX03_04075-DX03_04095, were detected; other fimbriae-coding genes are scattered throughout the genome.

### Chitinase, extracellular proteases, antibiotic and salinity resistance

*S. rhizophila* DSM14405^T^ is a biocontrol agent capable of synthesizing extracellular enzymes with anti-pathogenic activity such as chitinase and extracellular proteases, and is antagonistic against important fungal pathogens such as *Verticillium dahliae* and *Rhizoctonia solani* [11, 31]. In addition to its direct effect, *S. rhizophila* DSM14405^T^ is thought to also indirectly promote plant growth through biological control [14]. In the genome, Sr14405 DX03_17135 codes for a putative extracellular chitinase gene, and Sr14405s DX03_03415, DX03_05635, DX03_16295, DX03_17120 are predicted to code for extracellular proteases.

In general, *Stenotrophomona*s species are known to show resistance against a broad range of antibiotics [8, 31]. There are numerous resistance genes against various antibiotics in the genome of *S. rhizophila* DSM14405^T^, some code for general resistance, while others provide resistance against particular classes of antibiotics. The gene cluster extending from Sr14405 DX03_01540 to DX03_01550 was predicted to code for a multidrug export system. Another two multidrug resistance gene clusters were detected from Sr14405 DX03_02075 to DX03_02085 and from DX03_13460 to DX03_13470. A number of single multidrug resistance genes, such as *mdtN*, *mdtA* and Sr14405 DX03_03380 are scattered throughout the genome as well. Moreover, *macA* and *macB* code for the macrolide-specific efflux protein and a macrolide export ATP-binding/permease, respectively. Other identified genes include: Sr14405 DX03_06420 and *ampH* that code for ***β***-lactamases, Sr14405 DX03_08150 that codes for an aminoglycoside efflux pump, and a transposon tetracycline resistance gene (*tetX*).

*S. rhizophila* DSM14405^T^ possesses both *ggp*S and *ycaD*, which are essential for the synthesis and transport of the important osmolyte glucosylglycerol, which provides tolerance against salinity and salt stress [32]. Both *ggpS* and *ycaD* are absent in *S. maltophilia* R551-3 and K279a.

#### Surface polysaccharides

Homologs to *xan*A, *xanB,* and *rmlA*C were detected in *S. rhizophila* DSM14405^T^. These genes are involved in the biosynthesis of the *Xanthomonas* well-known surface polysaccharide xanthan, in biofilm formation [33] and the biosynthesis of lipopolysaccharides. It is noteworthy that *S. rhizophila* DSM14405^T^, similar to other members of the *Stenotrophomonas* species known so far, does not have a gum gene cluster and therefore cannot produce xanthan.

The bacterial capsule is an extracellular structure usually composed of polysaccharides which is considered an important virulence factor in surface adherence, antibiotic resistance, and preventing phagocytosis [34, 35]. Reckseidler et al. [36] demonstrated that the ability to synthesize capsule is crucial for virulence in the human pathogenic *Burkholderia*. In *S. rhizophila* DSM14405^T^, a gene block from Sr14405 DX03_11185 to DX03_11265 is homologous to a capsule biosynthesis gene cluster of *Pseudomonas pseudomallei*, described by [37]. This gene block includes genes that code for proteins of various functions such as signal transduction, transport, and biosynthesis of capsule polysaccharide components. None of the genes present in the *S. rhizophila* capsule gene block were detected in *S. maltophilia* R551-3 and K279a.

Alginate, an exopolysaccharide, is involved in the development and architecture of biofilms and protects bacteria from antibiotics and other antibacterial mechanisms [38–40]. Alginate biosynthesis genes *algI* and *algJ* code for the poly (beta-D-mannuronate) O-acetylase and the alginate biosynthesis protein, respectively. While both were detected in *S. rhizophila* DSM14405^T^, neither the plant-associated strain *S. maltophilia* R551-3 nor the human pathogenic *S. maltophilia* K279a contained either of these genes. *algI* is preceded by four genes which are also absent from both *S. maltophilia* R551-3 and K279a, with one of these being homologous to a gene coding for a cell morphology protein from the biocontrol agent *P. fluorescens* SBW25.

#### Secretion systems

While type II and V secretion system genes were identified in *S. rhizophila* DSM14405^T^, there is no type III secretion system present, as this is typical of *Stenotrophomonas*. Although there are several genes belonging to the type IV secretion system, a complete gene set was not detected in *S. rhizophila* DSM14405^T^. Furthermore, a gene block extending from Sr14405 DX03_08870 to DX03_09120 was identified in *S. rhizophila*, which includes numerous genes of the type VI secretion system (T6SS) including *icmF*, *impA*, genes belonging to the Hcp1 family, and genes coding for proteins with a T6SS Rhs element. With the exception of Sr14405 DX03_09050, DX03_09095, and DX03_09115, there were no homologs in *S. maltophilia* K279a and R551-3 to any of the genes of the *S. rhizophila* type VI secretion system block.

### One genus, two entirely different habitats and life styles? : Genome comparison between *S. rhizophila*DSM14405^T^and *S. maltophilia*K279a

All genes of the plant growth-promoting environmental *S. rhizophila* DSM14405^T^ and the clinical human pathogenic *S. maltophilia* K279a were compared. While absent from *S. maltophilia* K279a, numerous *S. rhizophila*-specific genes play a role in host-plant colonization. Some of these genes, as described earlier, are crucial for surface attachment, biofilm formation, secretion systems-driven molecular mechanisms, and tolerance of environmental stress such as high soil salinity. In addition, another *S. rhizophila*-specific gene was predicted to code for spermidine synthase (*speE*). Spermidine is a plant growth regulator and has been recently shown to strongly promote the growth of arugula plants [41]. There are also *S. rhizophila*-specific genes that are predicted to be involved in the biodegradation of bacterial and plant cell wall. m*ltD*, located closely to the *S. rhizophila* type VI secretion system gene block, codes for muramidase that plays an important role in the bacterial cell wall breakdown. Furthermore, a gene block stretching from Sr14405 DX03_09870 to DX03_09895 was predicted to code for several genes involved in the breakdown of plant cell walls.

As a next step, the *S. rhizophila* DSM14405^T^ having a counterpart in the plant-associated *S. maltophilia* R551-3, but with no homologous genes in the clinical *S. maltophilia* K279a were studied. Of these 88 genes, several help with the need to adapt to the plant and rhizosphere as the natural habitat. For instance, the endo-1,4-beta-xylanase B gene (*xynB*) is involved in plant cell wall biodegradation. Other genes conserved in *S. rhizophila* DSM14405^T^ and *S. maltophilia* R551-3 are the ferrichrome receptor genes, *fcuA* and *fhuA* which code for siderophore receptors, and the outer membrane adhesin-like gene (Sr14405 DX03_14745). Table 2 presents a list of selected *S. rhizophila* DSM14405^T^–specific genes with no homologs in the human pathogenic *S. maltophilia* K279a, together with their biological role. The complete list of the 884 *S. rhizophila* DSM14405^T^-specific genes which are not present in *S. maltophilia* K279a is provided in Additional file 2: Table S1.

**Table 2:** **Selected** ***S. rhizophila*** **DSM14405** ^**T**^ **–specific genes revealing no homologs in the human pathogenic** ***S. maltophilia*** **K279a with their role in coping with the environment and bacteria-plant interactions**

Bilogical role	Gene/Locus tag	(Putative) Product
Plant growth promotion	DX03_01135(*speE*)	Spermidine synthase
Secretion System	DX03_08905	lipoprotein
	DX03_08920	type VI secretion system effector
	DX03_08950	Rhs element Vgr protein with a type VI secretion system protein domain
	DX03_09065 (*icmf*)	type VI section system protein
	DX03_09080	type VI section system-associated protein ImpA family
	DX03_09090	Rhs element Vgr protein with a type VI secretion system protein domain
Bacterial and Plant cell wall breakdown	DX03_06125 *(mltD)*	Muramidase
	DX03_09870 *(xsa)*	xylosidase/arabinosidase
	DX03_09875 *(cbg1)*	beta-glucosidase
	DX03_09880	sialate O-Acetylesterase
	DX03_11570 *(aguA)*	alpha-glucuroidase
	DX03_20070 *(xynB)*	endo-1,4-beta-xylanase B
Resistance towards antibiotics and salinity	DX03_01540_42 (*mdtABC*)	three multidrug resistance proteins; form together a multidrug resistance protein channel
	DX03_06420	beta-lactamase
	DX03_06600 *(tetX)*	transposon tetracycline resistance protein
	DX03_06745 *(ampH)*	beta-lactamase
	DX03_08150	aminoglycoside efflux protein
	DX03_19375,*mdtA,mdtN*	multidrug resitance proteins
	DX03_19380	transposon tetracycline repressor protein
	DX03_04600 *(ggpS*)	glucoslglycerol-phosphate synthase; essential for the synthesis of the osmolyte glucoslglycerol
	DX03_04605 (*ycaD)*	glucoslglycerol transporter
Surface attachment and biofilm formation	DX03_11195 *(yccZ)*	capsule polysaccharide export protein
	DX03_11200 *(ymcC)*	Lipoprotein
	DX03_11240	capsule polysaccharide biosynthesis protein
	DX03_11265 (*wcaJ)*	colanic biosynthesis UDP-glucose lipid transferase
	DX03_14745	adhesin-like protein
	DX03_17015 (*algl)*	poly (beta-D-mannuronate) O-acetylase
	DX03_17020 (*algl)*	alginate bioynthesis protein Algl
Iron uptake	*fcuA,fhuA*	ferrichrome receptor proteins

**Figure 5:**
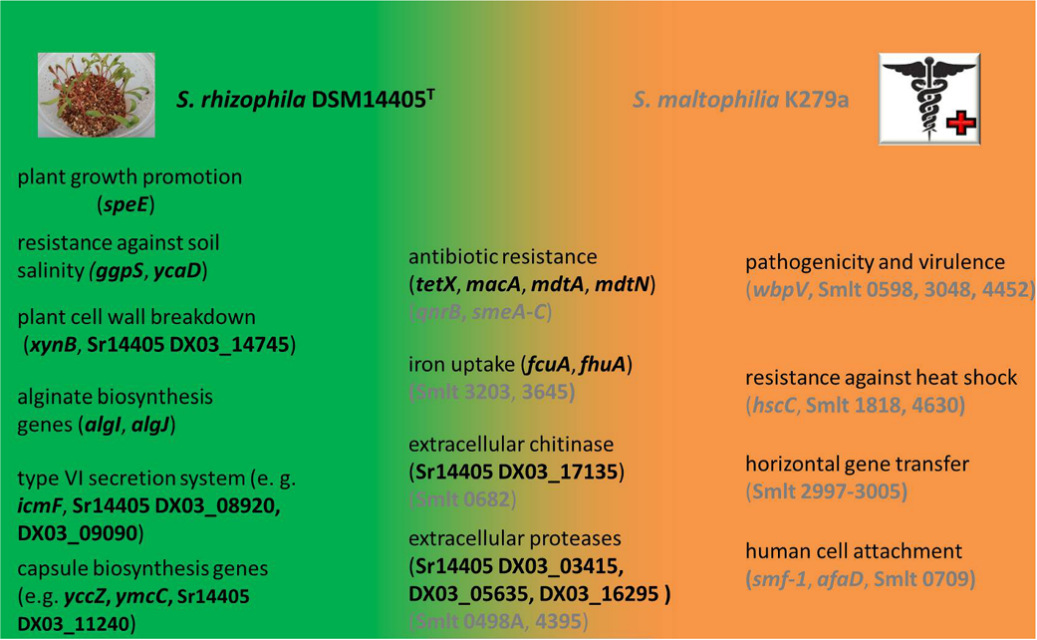
**Model showing the specific and shared mechanisms used by** ***S. rhizophila*** **and** ***S. maltophilia*** **K279a.** The plant growth promoting and biocontrol agent *S. rhizophila* DSM14405^T^ and the human-pathogenic clinical *S. maltophilia* K279a each use particular mechanisms that rely on species-specific genes to best adapt and perform in their habitats. The *S. rhizophila*-specific genes are shown in black while those genes in gray are specific to *S. maltophilia* K279a. Nevertheless, other crucial mechanisms such as ensuring access to biologically available iron and resistance against antibiotics are shared between both species (middle).

Next, the genomes of *S. rhizophila* DSM14405^T^ and *S. maltophilia* K279a were compared using the latter as the reference, which revealed that 1230 genes are specific to the human pathogenic K279a with no homologs in the plant-growth promoting *S. rhizophila*. While many of these genes are hypothetical or have unknown protein function, others play a specific role. Of the genes with a known or predicted function, many are involved in pathogenicity and virulence. For example, the gene block extending from Smlt 2997 to 3005 codes for proteins of the type IV secretion system, which is known to have a dual role of both horizontal gene transfer and pathogenicity. Other *S. maltophilia* K279a-specific virulence genes are Smlt 3048, 3683, and 4452, which were predicted to code for an outer membrane-located adhesin, hemolysin, and hemagluttinin, respectively. Furthermore, Smlt 4391, and *afaD* code for putative exopolysaccharide and adhesin, respectively. In addition, a K279a-specific putative fimbriae gene block (Smlt 706–709) codes for fimbrial adhesin proteins and their chaperones. Moreover, there are also several *S. maltophilia* K279a heat shock and chaperone-encoding genes that have no homologs in *S. rhizophila* DSM14405^T^, such as Smlt 1818 and Smlt 4629–4631, as both code for heat shock chaperone proteins. Synthesis of chaperones to cope with temperature-caused stress is fundamental for the natural habitat of *S. maltophilia* K279a as a human pathogenic strain. There are various *S. maltophilia* K279a-specific genes coding for antibiotic resistance and multidrug efflux pumps that are absent from *S. rhizophila* DSM14405^T^. For instance, *smeABC* (Smlt 4474–4476), which code for a multidrug efflux pump typical of *S. maltophilia* strains, is not present in DSM14405^T^. Nevertheless in total, six of the nine K279a genes that code for multidrug efflux pumps involved in antibiotic resistance [20] have a homolog in *S. rhizophila* DSM14405^T^. Other *S. maltophilia* K279a-specific genes with respect to antibiotic resistance include ***β***-lactamase genes, Smlt 4159 and 4211, and Smlt 1071 (*qnrB*), which codes for a fluoroquinolone resistance protein. Moreover, several heavy metal resistance genes for the transport of arsenate, mercuric, and copper are another genomic characteristic of the human pathogenic *S. maltophilia* K279a. Table 3 shows various *S. maltophilia* K279a genes with no homologs in the plant growth-promoting *S. rhizophila* DSM14405^T^ together with their products and biological roles. The complete list of the 1230 *S. maltophilia* K279a-specific genes is presented in additional file 3: Table S2. Figure 5 represents a model that summarizes crucial specific mechanisms harnessed by *S. rhizophila* DSM14405^T^ and *S. maltophilia* K279a to best adapt to their particular habitats together with the corresponding genes, and the important mechanisms that are shared between both species.

**Table 3:** **Selected human pathogenic** ***S. maltophilia*** **K279a–specific genes, which are involved in virulence and pathogenicity, and reveal no homologs in** ***S. rhizophila*** **DSM14405** ^**T**^

Bilogical role	Gene/Locus tag	(Putative) Product
Virulence	smf-1,0709	fimbrial adhesin proteins
	0707	pili chaperone protein
	mrkC	outer membrane usher protein
	3048	Hep Hag family adhesin
	wbpV (3683)	hemolysin protein
	4391	exopolysaccharide synthesis protein
	afaD	Non-fembrial adhesin
	4452	cell surface haemagluttinin protein
Secretion system-mediated pathogenicity, horizontal gene transfer	2997,3000	type IV secretion system transmembrane proteinS
	2999,3002,3003	type IV secretion conjugal transfer proteins
	3005	VirB9 protein
Heat shock resistance, chaperones	1818	heat shock chaperone protein
	hscC	chaperone heat shock Hsp70 protein
	4630,4631	heat shock chaperone protein
Antibiotic resistance	qnrB	flouroquinolone resistance protein
	2642	macrolide-specific ABC-type efflux
	4159,4211	beta-lactamase
	smeC (oprM)	multidrug efflux system outer membrane protein
	smerB	multidrug efflux protein
	smeA (acrA)	drug resistance efflux protein

### The temperature limit: the transcriptional response of *S. rhizophila*DSM14405^T^to 35°C

**Figure 6:**
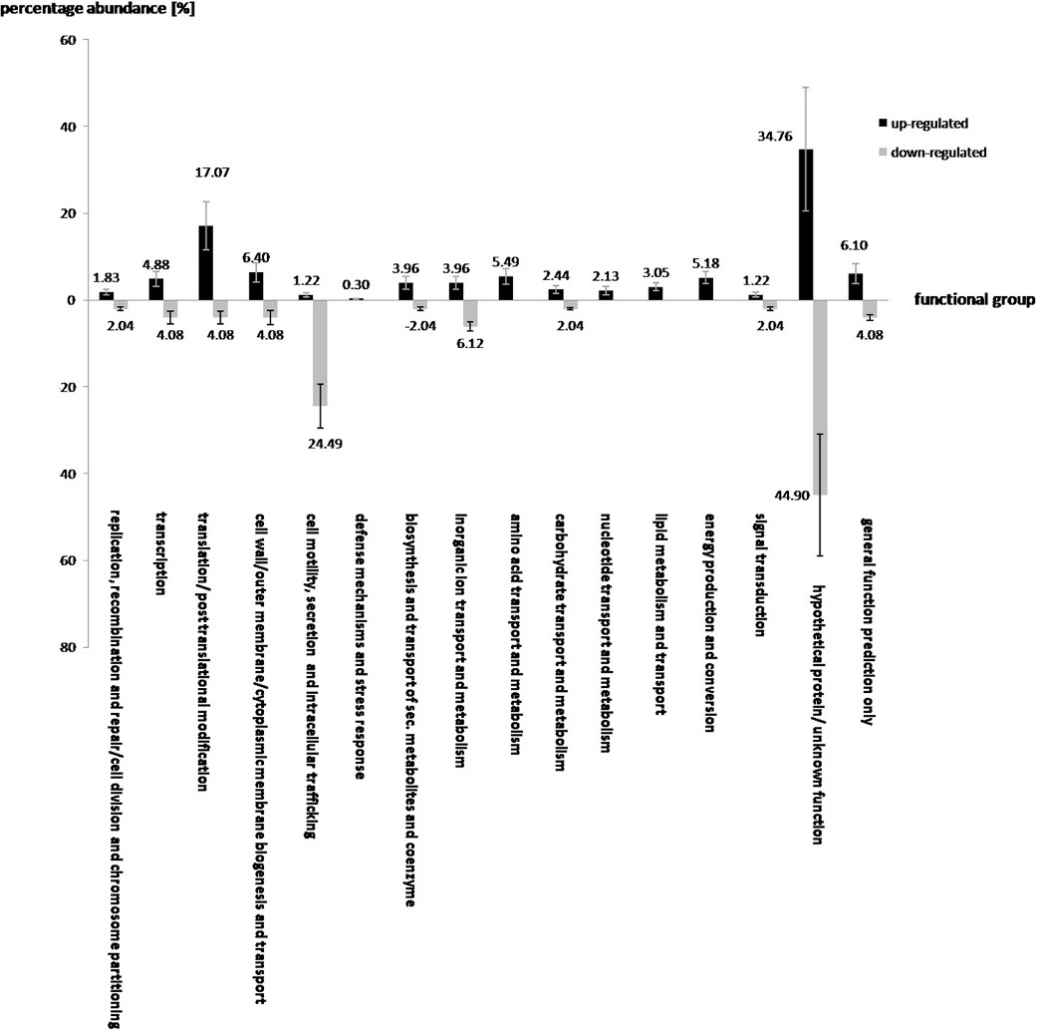
**The impact of heat shock (35°C) on the gene expression of** ***S. rhizophila*** **DSM14405** ^**T**^ **.** Genes responsible for translation and post-translational modification, the metabolism and transport of amino acids, nucleotides, lipids, and the production and conversion of energy are up-regulated while genes involved in cell motility and intracellular trafficking are strongly down-regulated. The values above each column correspond to the percentage abundance of the corresponding functional gene group relative to the total count of the up and down-regulated genes. The transcription fold change for each CDS corresponds to the ratio calculated for *S. rhizophila* treated with 35°C compared to 30°C. Data are presented as the mean value of two independent replicates. The error bar shown on each functional group corresponds to the mean value of errors for all genes belonging to that functional group.

In contrast to other *Stenotrophomonas* strains including *S. maltophilia* K279a and R551-3, *S. rhizophila* DSM14405^T^ is not able to grow at 37°C, which is a critical temperature for strains to colonize the human body. To understand why this strain and other *S. rhizophila* strains cannot grow at this temperature and to reveal the underlying molecular mechanisms behind, the impact of high temperature (35°C) on *S. rhizophila* DSM14405^T^ was studied using a transcriptomic approach. In comparison with growth at 30°C, 328 and 49 genes are significantly up and down-regulated, respectively, as the result of a severe heat shock of 35°C (Figure 6). As presented, genes responsible for translation and post-translational modification along with other functional gene groups including those involved in the metabolism and transport of amino acids, nucleotides, lipids, and the production and conversion of energy are up-regulated at 35°C. Moreover, those genes whose functions are only generally known are also mostly up-regulated suggesting that, in *S. rhizophila* DSM14405^T^, a significant number of the genes involved in the response to severe heat shock code for proteins with unknown function. In contrast, genes responsible for cell motility, secretion and intracellular trafficking show a pronounced down-regulation at 35°C, making up 24.49% of all down-regulated genes. Of the genes with a significant transcription fold change as the result of the shift from 30 to 35°C, some code for products with known biological function (Table 4). As shown in the table, genes coding for chaperones involved in both general stress and heat shock specific response including *dnaJ-K*, and *groS-L and htpG*, respectively, are up-regulated at 35°C. Other up-regulated genes include *rpoH* which codes for the heat shock specific sigma factor, and numerous genes coding for proteases that are responsible for the degradation of stress-denatured proteins. Furthermore, genes responsible for cell suicide mechanisms, Sr14405 DX03_18795 and DX03_18990, are also up-regulated as the result of the shift from 30 to 35°C. In contrast, as shown in Table 4 genes coding for pili-driven cell motility are negatively affected at 35°C.

**Table 4:** **Selected** ***S. rhizophila*** **DSM14405** ^**T**^ **genes with known biological roles impacted by the 35°C heat shock**

Gene/Locus tag	(Putative) Product	Transcription fold change	Biological role
DX03_01700 (*rpoH*)	sigma 32	3.0	heat shock sigma factor
DX03_05495	negative regulator of sigma 24	3.1	transcription regulation
DX03_11865 (*dnaJ*)	chaperone DnaJ	3.1	stress response
DX03_11870 (*dnaK*)	chaperone DnaK	2.9	stress response
DX03_11875 (*grpE*)	chaperone complex protein	2.6	prevents the aggregation of stress-denatured proteins
DX03_02015 (*groS*)	co-chaperonin GroES	2.4	heat shock chaperone
DX03_02020 (*groL*)	chaperonin GroEL	1.9	heat shock chaperone
DX03_12190 (*htpG*)	heat shock chaperone	2.0	heat shock response
DX03_07545 (*fkpA*)	chaperone	2.0	stress response
DX03_10250 (*clpA*)	ATPase with chaperone activity	1.8	stress response
DX03_14890 (*yraA*)	protease	2.3	degradation of abnormal/stress-denatured proteins
DX03_05505 (*degP*)	protease	2.1	degradation of abnormal/stress-denatured proteins
DX03_02875 (*hslV*)	protease	1.5	degradation of abnormal/stress-denatured proteins
DX03_06825 (*htpX*)	protease	1.5	degradation of abnormal/stress-denatured proteins due to heat shock
DX03_15635 (*lon*)	Lon Protease	1.5	degradation of abnormal/stress-denatured proteins
DX03_03030 (*prc*)	protease	1.5	Protein degradation under thermal stress
DX03_05315 (*cbpA*)	DNA-binding protein; accessory protein for DnaJ	1.8	heat shock response
DX03_11520 (*gtaB*)	UTP--glucose-1-phosphate uridylyltransferase	1.6	general stress protein
DX03_18990	killer protein	2.4	suicide mechanism
DX03_18795	entericidin EcnAB	3.3	suicide mechanism
DX03_06640	sigma 24	0.3	primary general stress sigma factor
DX03_04075	fimbrial assembly family protein	0.6	pili-driven motility
DX03_04080	pilus assembly protein PilO	0.6	pili-driven motility
DX03_04085	pilus assembly protein PilP	0.6	pili-driven motility
DX03_04090 (*pilQ*)	pilus assembly protein PilQ	0.6	pili-driven motility
DX03_04405 (*fimA*)	fimbrial protein	0.6	pili-driven motility
DX03_04895 (*pilJ*)	fimbrial protein	0.6	pili-driven motility
DX03_10405 (*fliC*)	A-type flagellin	0.5	pili-driven motility
DX03_12885 (*ppdD*)	fimbrial protein pilin	0.4	pili-driven motility
DX03_12890	type 4 fimbrial biogenesis	0.6	pili-driven motility
DX03_12905	pre-pilin leader sequence	0.5	pili-driven motility
DX03_12910	pre-pilin leader-like sequence	0.5	pili-driven motility

Fold changes correspond to the *S. rhizophila* DSM14405^T^ treated with 35°C compared to 30°C (control).

## Discussion

In the present work, we characterized the genomic features of the plant growth promoting and biocontrol agent *S. rhizophila* DSM14405^T^. In addition, the comparison between the genome of *S. rhizophila* with other environmental and clinical *Stenotrophomonas* model strains, *S. maltophilia* R551-3 and K279a, respectively revealed a great deal of homology shared among all three bacteria. Surprisingly, the comparison of *S. rhizophila*’s genes with either of the other two strains showed that the total number of genes shared between *S. rhizophila* and the human-pathogenic *S. maltophilia* K279a is only slightly less than the total gene number shared with the plant-associated beneficial *S. maltophilia* R551-3 strain. Given this significant degree of similarity, we concluded that the immense difference between *S. rhizophila* and *S. maltophilia* K279a with regard to the habitat and lifestyle must be based upon the role of those genes that are specific to either of the strains and absent from the other. On this account, a number of *S. rhizophila* specific genes coding for products of known function were detected and classified according to their biological roles. These include genes involved in plant growth promotion, plant cell wall degradation, biofilm formation, resistance against salinity and the type VI secretion system (T6SS).

Clearly as an environmental plant-associated strain, *S. rhizophila* faces particular habitat-specific factors such as those that impose some sort of osmotic stress on the cell including root exudates and salinity. In a study published recently, we used a transcriptomic approach to study the manner of response of *S. rhizophila* DSM14405^T^ to osmotic stress [42]. Surprisingly, the combination of both the genomic and transcriptomic data reveals that a significant number of the genes with known biological role that are greatly involved in osmotic stress protection including *cbg1*, *xynB*, *ggpS*, *ycaD*, *algJ* and the T6SS are specific to *S. rhizophila* and absent from *S. maltophilia* K279a. This finding confirms the importance of the strain specific genes in the adaptation and performance of *S. rhizophila* in its natural habitat. As a clinical human-pathogenic strain, *S. maltophilia* K279a also possesses particular genetic potentials that make possible its lifestyle (Table 3, Figure 5). The role of type IV secretion system, hemolysin and other adherence components in virulence and pathogenicity was described earlier. A yet more crucial feature of *S. maltophilia* K279a with regard to performance in its habitat, however, is perhaps its ability to grow at 37°C. Also in this regard, this strain possesses specific heat shock resistance genes that are absent from *S. rhizophila*.

As mentioned earlier, *S. rhizophila* DSM14405^T^ is not able to grow at 37°C. To understand the manner of response of *S. rhizophila* to severe growth-inhibiting temperature, a genome-wide gene expression analysis was performed at 35°C using transcriptomics. The positive impact of the growth temperature at 35°C compared to 30°C on several functional gene groups including those responsible for the metabolism and transport of amino acids, lipids and the energy providing genes is due to the heat stress that the cell is coping with. Furthermore, studying the expression of single genes revealed that those genes coding for general and heat shock specific chaperones, various proteases that break down stress-denatured, abnormal proteins are up-regulated. For example at 35°C, the primary general stress sigma factor 24 coding gene, which is strongly expressed at an earlier stage under cell stress, and initiates the expression of *rpoH* (the heat shock specific sigma factor 32), specific chaperones (e. g. *fkpA*) and protease coding genes (e. g. *degP*) is down-regulated while *rpoH*, and other response mechanisms show up-regulation. This confirms an advanced state of shock for *S. rhizophila* at 35°C, at which heat shock specific response mechanisms are strongly activated. In this regard, the up-regulation of Sr14405 DX03_18795 and DX03_18990 (absent from *S. maltophilia* K279a) which code for toxin/antidote-based suicide systems further illustrate the mechanisms used by *S. rhizophila* DSM14405^T^ to respond to the severe heat stress. Moreover, reduced cell motility, which is also part of these response mechanisms, is presumably due to the fact that costly processes such as motility are to be minimized during harsh stress times.

## Conclusions

Overall, there is a great deal of similarity between the beneficial and human-pathogenic *Stenotrophomonas* model strains. Nevertheless, the adaptation to the habitat and lifestyle to guarantee the survival of the species is cared for by mechanisms specific to either of the groups, and many genes underlying these mechanisms in *S. rhizophila* DSM14405^T^ are strain-specific. Furthermore according to the genomic and transcriptomic analyses together with its physiological characteristics, *S. rhizophila* poses no threat to human health and could hence be safely applied in biotechnology.

## Methods

### Bacterial strain

*S. rhizophila* DSM14405^T^ (syn. e-p10 and p69) was isolated from the rhizosphere of oilseed rape in Rostock, Germany [11, 31].

### Genome sequencing, assembly, annotation of *S. rhizophila*DSM14405^T^

The genome of *S. rhizophila* DSM14405^T^ was sequenced using a combination of next generation sequencing platforms. A first draft assembly based on 905,689 reads of an 8kbp paired-end library (Roche 454 GS, FLX Titanium, Helmholtz Center Munich, Germany) with a total of 167.1 Mbps (36-fold coverage) was generated with Newbler 2.6 (Roche Diagnostics, Penzberg, Germany). This assembly consisted of 175 contigs, 122 of which could be joined into a single circular scaffold. Gaps resulting from repetitive sequences were resolved by *in silico* gap filling, remaining gaps were closed by PCR followed by Sanger sequencing or by long reads from a Pacific BioSciences sequencing run (PacBio RS, 150,305 reads, 174.8 Mbps, 38-fold coverage, GATC, Konstanz, Germany), yielding a draft genome of 4,648,936 bps. To improve the quality of the sequence by eliminating 454 sequencing artefacts in homopolymer stretches, the genome was subsequently sequenced using the Illumina paired-end method (Illumina HiSeq 2000, 15,086,654 reads, 1508 Mbp; 324-fold coverage, Ambry Genetics, Aliso Viejo, CA, USA). The Illumina reads were aligned to the draft genome with CLC Genomics Workbench 4.7.2 (CLC bio, Aarhus, Denmark). The final consensus sequence was derived by counting instances of each nucleotide at a position and then letting the majority decide the nucleotide in the consensus sequence. Genes were identified with the Prodigal gene finder [43], ARAGORN [44], and RNAmmer 1.2 [45]. Functional annotation of the predicted genes was performed using BASys [46], which provides annotations with respect to Clusters of Orthologous Groups (COG) [47], Pfam [48] and Gene Ontology (GO) [49]. The final genome includes 4,648,976 bases with a GC content of 67.26%.

#### Comparative genomics and bioinformatic analyses

Whole genome comparisons between *S. rhizophila* DSM14405^T^, *S. maltophilia* R551-3 and K279a were performed using Mauve 2.3 [50] and Artemis Comparison Tool (ACT) [51]. In the approach using Mauve, the Progressive Mauve algorithm was used to score the genome alignment. DNAPlotter [52] was used for circular genome visualization. Orthologous coding DNA sequences (CDS) shared between *S. rhizophila* DSM14405^T^ and the other two *Stenotrophomonas* were assessed by performing reciprocal BLASTp best hits with an identity and e-value threshold of 30% and 10^-6^, respectively.

### Cell culture growth conditions for transcriptomic analyses

*S. rhizophila* DSM14405^T^ was grown in 100 ml Erlenmeyer flasks containing 50 ml CAA minimal medium at 30°C under agitation until OD_600_ of 0.9 was reached. To introduce heat shock prior to RNA extraction, some of the flasks containing the culture were exposed to 35°C for 2 h under identical agitation conditions while the rest of the flasks remained at 30°C for the same period of time (control).

### RNA extraction and transcriptomic analyses

RNA was extracted from 250 ***μ***l of each of the cultures exposed to 30°C and 35°C in duplicates using the RNeasy Mini Kit and the RNAprotect® Bacteria Reagent according to the manufacturer’s protocol (Qiagen, Hilden, Germany). Total RNA was sent to a sequencing service offered by GATC Biotech (Konstanz, Germany) where the samples were processed according to company’s proprietary protocols including depletion of rRNA (Ribo-Zero RNA Removal Kit, Epicentre, Madison, USA), fragmentation of mRNA, random-primed synthesis of cDNA, double strand synthesis and library preparation. Sequencing was performed using Illumina HiSeq 2000 and 50 bp single read mode resulting in 15,202,000 to 37,966,600 quality reads per sample. Reads were mapped to the reference genome of *S. rhizophila* DSM14405^T^ and only the corresponding normalized values for the reads that uniquely mapped to each CDS were used to assess the changes in gene transcription. The transcription fold change as the result of the temperature shift was assessed for each CDS by dividing its read number for the cell culture treated at 35°C by the value from the culture grown at 30°C. Of the total genes either up or down-regulated, only those showing fold changes greater than or equal to 1.5 and less than or equal to 0.6 were considered as significantly impacted.

### Availability of supporting data

The genome of *Stenotrophomonas rhizophila* DSM14405^T^ was deposited in the NCBI database and can be accessed under the BioProject Nr. CP007597.

## Electronic supplementary material

Additional file 1: Figure S1: Genome-scale comparison between the three strains using Artemis Comparison Tool (ACT). (JPEG 167 KB)

Additional file 2: Table S1: The list of the 884 *S. rhizophila* DSM14405^T^ specific genes that are absent from *S. maltophilia* K279a. (PDF 51 KB)

Additional file 3: Table S2: The list of the 1230 *S. maltophilia* K279a specific genes that are absent from *S. rhizophila* DSM14405^T^. (PDF 64 KB)
