# Supplementary figures and images for: Stenotrophomonas comparative genomics reveals genes and functions that differentiate beneficial and pathogenic bacteria
Source: BMC Genomics. 2014 Jun 18;15(1):482. doi: 10.1186/1471-2164-15-482 (PMC4101175; doi:10.1186/1471-2164-15-482)

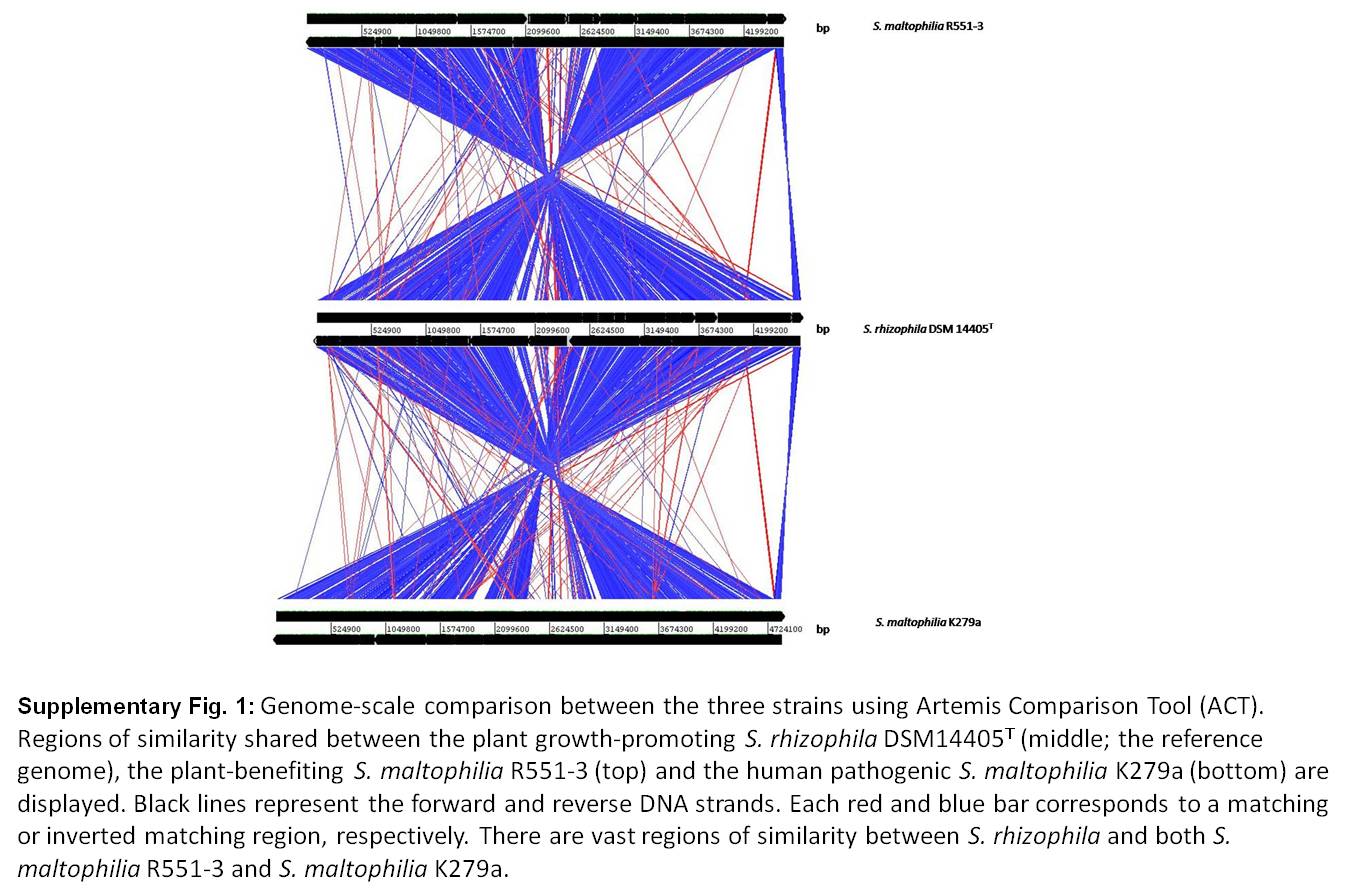

Supplement: Supplementary file 1 — Additional file 1: Figure S1: Genome-scale comparison between the three strains using Artemis Comparison Tool (ACT). (JPEG 167 KB) [file 12864_2013_6236_MOESM1_ESM.jpeg]
