# Supplementary material for: Stenotrophomonas comparative genomics reveals genes and functions that differentiate beneficial and pathogenic bacteria
Source: BMC Genomics. 2014 Jun 18;15(1):482. doi: 10.1186/1471-2164-15-482 (PMC4101175; doi:10.1186/1471-2164-15-482)
Supplement: Supplementary file 2 — Additional file 2: Table S1: The list of the 884 S. rhizophila DSM14405T specific genes that are absent from S. maltophilia K279a. (PDF 51 KB) [file 12864_2013_6236_MOESM2_ESM.pdf]

Supplementary Table 1 : The list of the 884 *S. rhizophila* DSM14405T specific genes that are absent from *S. maltophilia* K279a

Nr.	locus tag	product
1	1023	Hypothetical
2	1025	Transglycosylase-Associated Protein
3	1030	Mitomycin resistance protein mcrB
4	1036	Hypothetical Protein 1036
5	1040	Hypothetical
6	1042	Hypothetical
7	1050	Hypothetical
8	1053	Hypothetical
9	1054	Hypothetical Protein 1054
10	1055	Nodulation protein L
11	1057	Hypothetical Protein 1057
12	1063	Hypothetical Protein 1063
13	1068	Hypothetical
14	1093	Hypothetical Protein 1093
15	1097	Hypothetical Protein 1097
16	1105	Hypothetical Protein 1105
17	1111	Hypothetical Protein 1111
18	1124	Hypothetical Protein 1124
19	1125	Hypothetical
20	1140	Hypothetical Protein 1140
21	1141	Hypothetical Protein 1141
22	1143	Hypothetical
23	1144	Hypothetical
24	1169	Hypothetical
25	1172	Hypothetical
26	1193	SapC Family Protein
27	1194	Hypothetical Protein 1194
28	1195	Hypothetical Protein 1195
29	1198	HsdR Family Type I Site-Specific Deoxyribonuclease
30	1199	Uncharacterized adenine-specific methylase MJ1220
31	1200	Hypothetical
32	1201	Hypothetical
33	1202	Hypothetical Protein 1202
34	1203	Hypothetical Protein 1203
35	1204	Hypothetical

36	1205	LysR Family Transcriptional Regulator
37	1206	Hypothetical
38	1207	Hypothetical Protein 1207
39	1212	XRE Family Transcriptional Regulator
40	1213	Modification methylase TaqI
41	1214	Nodulation protein V
42	1215	Hypothetical
43	1216	Hypothetical Protein 1216
44	1217	Hypothetical
45	1218	Hypothetical Protein 1218
46	1219	Hypothetical Protein 1219
47	1221	Hypothetical
48	1226	Hypothetical
49	1243	Hypothetical Protein 1243
50	1244	RNA polymerase sigma factor sigW
51	1255	MarR Family Transcriptional Regulator
52	1258	Hypothetical
53	1259	Hypothetical
54	1260	Hypothetical
55	1275	Hypothetical Protein 1275
56	1276	Hypothetical Protein 1276
57	1279	Hypothetical Protein 1279
58	1280	Hypothetical
59	1307	Ferrichrome receptor fcuA
60	1323	Transcription Factor Jumonji Domain-Containing Protein
61	1324	NB-Dependent Receptor
62	1325	Glucose/galactose transporter
63	1327	Periplasmic beta-glucosidase
64	1335	Hypothetical
65	1336	Hypothetical
66	1337	Hypothetical
67	1338	Hypothetical
68	1341	Hypothetical
69	1342	Hypothetical
70	1344	Hypothetical
71	1359	Hypothetical
72	1366	D-aminopeptidase
73	1367	Probable phosphoenolpyruvate synthase

74	1369	Hypothetical
75	1370	Hypothetical
76	1371	Hypothetical
77	1372	Hypothetical
78	1373	ThiJ/PfpI Domain-Containing Protein
79	1374	Helix-Turn-Helix Domain-Containing Protein
80	1375	HTH-type transcriptional regulator ptxR
81	1376	NAD(P)H azoreductase
82	1377	Hypothetical Protein 1377
83	1378	Hypothetical
84	1387	Transcription regulatory protein opdE
85	1388	Uncharacterized protein PA2218
86	1389	Putative transcriptional regulator
87	1390	Hypothetical
88	1391	Hypothetical Protein 1391
89	1396	Arylamine N-acetyltransferase
90	1405	Glycine cleavage system transcriptional activator
91	1406	Probable disulfide bond reductase yfcG
92	1407	Uncharacterized protein Rv0953c/MT0980
93	1408	Hypothetical Protein 1408
94	1413	Hypothetical Protein 1413
95	1414	Multidrug resistance protein MdtC
96	1415	Multidrug resistance protein MdtB
97	1416	Multidrug resistance protein mdtA
98	1422	Hypothetical
99	1423	Hypothetical Protein 1423
100	1424	Hypothetical Protein 1424
101	1427	Hypothetical Protein 1427
102	1434	Hypothetical Protein 1434
103	1459	Hypothetical Protein 1459
104	1460	Putative kinase YjjJ
105	1468	UPF0187 protein yneE
106	1469	Hypothetical
107	1472	Hypothetical
108	1473	Uncharacterized HTH-type transcriptional regulator yafC
109	1474	Transcriptional Regulator
110	1475	Hypothetical Protein 1475
111	1476	TetR-Family Transcriptional Regulator

112	1477	Hypothetical Protein 1477
113	1478	Multidrug resistance protein mdtN
114	1479	Hypothetical
115	1480	Uncharacterized protein YmdC
116	1481	Hypothetical Protein 1481
117	1482	Hypothetical
118	1485	Filamentation Induced By CAMP Protein Fic
119	1486	Hypothetical Protein 1486
120	1487	Hypothetical Protein 1487
121	1490	Pesticin receptor
122	1491	AraC Family Transcriptional Regulator
123	1495	Antibiotic Biosynthesis Monooxygenase
124	1496	Putative sulfate transporter ybaR
125	1500	Hypothetical Protein 1500
126	1508	Hypothetical
127	1516	Hypothetical
128	1528	Chorismate Mutase
129	1529	Hypothetical Protein 1529
130	1541	Hypothetical Protein 1541
131	1544	Uncharacterized protein HI_1456
132	1552	Benzaldehyde dehydrogenase [NAD+]
133	1553	Aryl-alcohol dehydrogenase
134	1554	Fis Family Transcriptional Regulator
135	1559	Virulence-associated protein I
136	1563	Hypothetical Protein 1563
137	1564	Hypothetical Protein 1564
138	1584	Diguanylate cyclase DosC
139	1588	Uncharacterized protein HI_1246
140	1628	Hypothetical Protein 1628
141	1630	Biopolymer transport protein exbD2
142	1647	Uncharacterized HTH-type transcriptional regulator ybcM
143	1653	Hypothetical Protein 1653
144	1658	Hypothetical
145	1659	Hypothetical
146	1660	Hypothetical
147	1665	RIO-type serine/threonine-protein kinase Rio2
148	1685	Hypothetical Protein 1685
149	1691	Hypothetical

150	1712	Magnesium transporter mgtE
151	1713	Conserved Hypothetical Protein
152	1719	Hypothetical Protein 1719
153	1720	NADP-specific glutamate dehydrogenase
154	1740	Hypothetical
155	1741	Hypothetical
156	1753	Hypothetical
157	1761	Hypothetical Protein 1761
158	1762	Hypothetical Protein 1762
159	1765	Uncharacterized protein yiiG
160	1780	Hypothetical
161	1796	Hypothetical Protein 1796
162	1800	Hypothetical
163	1801	Hypothetical Protein 1801
164	1807	Hypothetical Protein 1807
165	1808	Hypothetical
166	1809	Hypothetical
167	1825	Hypothetical
168	1843	Aminopeptidase N
169	1845	Hypothetical Protein 1845
170	1855	Hypothetical
171	1856	Putative sodium/proton-dependent alanine carrier protein yrbD
172	1867	Hypothetical Protein 1867
173	1868	Hypothetical Protein 1868
174	1878	Probable spermidine synthase
175	1886	Transposase
176	1891	Uncharacterized protein Rv0899/MT0922
177	1898	Hypothetical Protein 1898
178	1899	Hypothetical Protein 1899
179	1904	Methylamine utilization protein MauG
180	1905	NB Dependent Receptor
181	1906	Uncharacterized protein yddB
182	1911	AraC Family Transcriptional Regulator
183	1917	Glucosylglycerol-phosphate synthase
184	1918	Uncharacterized MFS-type transporter CKO_02171
185	1922	Hypothetical
186	1934	LA Protein
187	1940	Hypothetical

188	1941	Hypothetical
189	1942	Hypothetical Protein 1942
190	1943	Hypothetical Protein 1943
191	1944	Hypothetical
192	1945	Hypothetical Protein 1945
193	1947	Peptidase
194	1948	Hypothetical
195	1949	Hypothetical Protein 1949
196	1950	Putative lipoprotein lprI
197	1953	Hypothetical Protein 1953
198	1954	Hypothetical
199	1960	Hypothetical Protein 1960
200	1966	dTDP-rhamnosyl transferase rfbF
201	1991	Hypothetical Protein 1991
202	1993	Alcohol dehydrogenase [acceptor]
203	2013	Hypothetical Protein 2013
204	2014	Hypothetical Protein 2014
205	2015	Conserved Hypothetical Protein
206	2016	Hypothetical Protein 2016
207	2017	Hypothetical Protein 2017
208	2018	Hypothetical Protein 2018
209	2019	Hypothetical Protein 2019
210	2020	Hypothetical Protein 2020
211	2021	Hypothetical
212	2022	Hypothetical Protein 2022
213	2032	Autoinducer 2 sensor kinase/phosphatase luxQ
214	2033	Autoinducer 2 sensor kinase/phosphatase luxQ
215	2034	Sensory/regulatory protein RpfC
216	2045	ATP Dependent DNA Ligase
217	2050	Hypothetical
218	2064	Hypothetical
219	2065	Hypothetical
220	2067	Hypothetical
221	2083	Hypothetical
222	2102	Hypothetical
223	2116	Hypothetical Protein 2116
224	2117	Hypothetical Protein 2117
225	2127	Hypothetical

226	2129	Hypothetical Protein 2129
227	2154	Hypothetical
228	2202	Uncharacterized peptidase yuxL
229	2226	Hypothetical
230	2227	Inner membrane protein yqiK
231	2228	Hypothetical
232	2229	Signal Recognition Particle-Docking Protein FtsY
233	2230	Protein klaB
234	2267	Transcriptional Regulatory Protein-Like Protein
235	2268	6-aminohexanoate-dimer hydrolase
236	2280	Hypothetical Protein 2280
237	2281	Hypothetical
238	2282	Hypothetical
239	2285	Hypothetical Protein 2285
240	2287	Homoserine/homoserine lactone efflux protein
241	2288	3-methyl-2-oxobutanoate hydroxymethyltransferase
242	2289	Hypothetical Protein 2289
243	2290	Hypothetical Protein 2290
244	2293	Conserved Hypothetical Protein
245	2294	Hypothetical Protein 2294
246	2295	Hypothetical Protein 2295
247	2301	MarR Family Transcriptional Regulator
248	2302	Tetracycline resistance protein from transposon Tn4351/Tn4400
249	2309	Hypothetical Protein 2309
250	2312	Anti-FecI Sigma Factor FecR
251	2313	Amidohydrolase
252	2314	Aminobenzoyl-glutamate utilization protein B
253	2316	Hypothetical Protein 2316
254	2317	Uncharacterized protein yjiJ
255	2319	Hypothetical
256	2320	Hypothetical Protein 2320
257	2323	Hypothetical Protein 2323
258	2324	Hypothetical Protein 2324
259	2325	Chemoreceptor mcpA
260	2327	Hypothetical Protein 2327
261	2329	D-alanyl-D-alanine carboxypeptidase
262	2342	Hypothetical Protein 2342
263	2346	Hypothetical Protein 2346

264	2384	Hypothetical Protein 2384
265	2385	Hypothetical Protein 2385
266	2386	Hypothetical
267	2388	Hypothetical Protein 2388
268	2389	Hypothetical Protein 2389
269	2417	Hypothetical
270	2437	Hypothetical Protein 2437
271	2438	Hypothetical Protein 2438
272	2441	Thiol-disulfide oxidoreductase resA
273	2466	Hypothetical
274	2473	UPF0053 protein yugS
275	2487	Amidohydrolase
276	2501	Uncharacterized oxidoreductase YajO
277	2528	Hypothetical Protein 2528
278	2531	Hypothetical Protein 2531
279	2534	Hypothetical Protein 2534
280	2537	Uncharacterized NTE family protein ylbK
281	2539	Hypothetical Protein 2539
282	2541	Uncharacterized protein yfhB
283	2545	Glyoxalase/Bleomycin Resistance Protein/Dioxygenase
284	2547	Hypothetical Protein 2547
285	2549	Restriction Endonuclease
286	2550	Conserved Hypothetical Protein
287	2557	Hypothetical Protein 2557
288	2572	Hypothetical
289	2574	Virulence Regulator
290	2575	HTH-type transcriptional regulator AlkR
291	2576	Purine ribonucleoside efflux pump nepI
292	2577	Signal Transduction Histidine Kinase LytS
293	2579	Acyl Co A Thioester Hydrolase
294	2580	Hydroxymethylpyrimidine/phosphomethylpyrimidine kinase
295	2581	Abortive Infection Protein
296	2585	Uncharacterized protein y4lL
297	2586	Hypothetical Protein 2586
298	2587	Hypothetical
299	2589	Hypothetical
300	2590	Hypothetical
301	2591	Hypothetical Protein 2591

302	2592	Hypothetical Protein 2592
303	2593	Hypothetical
304	2594	Zinc-type alcohol dehydrogenase-like protein SAV2186
305	2595	Serine 3-dehydrogenase
306	2596	N-ethylmaleimide reductase
307	2597	TetR Family Transcriptional Regulator
308	2598	TetR Family Transcriptional Regulator
309	2599	RND Family Efflux Transporter MFP Subunit
310	2600	Probable aminoglycoside efflux pump
311	2601	3-oxoacyl-[acyl-carrier-protein] synthase 2
312	2602	Hypothetical Protein 2602
313	2603	Hypothetical Protein 2603
314	2605	Hypothetical
315	2606	Hypothetical Protein 2606
316	2607	Uncharacterized HTH-type transcriptional regulator yqhC
317	2608	Uncharacterized oxidoreductase MexAM1_META1p0182
318	2609	Hypothetical
319	2610	Saccharopine Dehydrogenase
320	2611	Hypothetical
321	2613	Uncharacterized MFS-type transporter ytbD
322	2614	Hypothetical
323	2615	Hypothetical Protein 2615
324	2617	Uncharacterized MFS-type transporter Rv2456c/MT2531
325	2623	Hypothetical Protein 2623
326	2624	Hypothetical
327	2625	NB-Dependent Receptor
328	2627	UPF0337 protein XCC0070
329	2629	Hypothetical Protein 2629
330	2631	RsbT co-antagonist protein rsbRA
331	2632	RsbT antagonist protein rsbS
332	2633	Serine/threonine-protein kinase rsbT
333	2634	Stage II Sporulation Protein E
334	2639	UPF0361 protein yoaM
335	2641	Probable ATP-dependent helicase lhr
336	2642	Hypothetical Protein 2642
337	2645	Hypothetical Protein 2645
338	2647	GAF Sensor Signal Transduction Histidine Kinase
339	2648	Hypothetical

340	2664	PspC Domain Protein
341	2674	Hypothetical Protein 2674
342	2684	Hypothetical Protein 2684
343	2689	Hypothetical Protein 2689
344	2690	Hypothetical Protein 2690
345	2691	Glutathione S-transferase GST-6.0
346	2693	Hypothetical Protein 2693
347	2696	Hypothetical Protein 2696
348	2697	Hypothetical Protein 2697
349	2701	Hypothetical Protein 2701
350	2703	Hypothetical Protein 2703
351	2715	Uncharacterized HTH-type transcriptional regulator Rv3095/MT3179
352	2720	Hypothetical Protein 2720
353	2722	Hypothetical
354	2723	N-ethylmaleimide reductase
355	2726	Glyoxalase/Bleomycin Resistance Protein/Dioxygenase
356	2732	Hypothetical
357	2733	Hypothetical Protein 2733
358	2734	Hypothetical
359	2735	Muramidase-2
360	2736	Hypothetical Protein 2736
361	2737	Rhs Element Vgr Protein
362	2738	Hypothetical Protein 2738
363	2739	Conserved Hypothetical Protein
364	2740	Hypothetical
365	2741	Rhs Element Vgr Protein
366	2742	Hypothetical
367	2743	Hypothetical
368	2744	Hypothetical
369	2745	Hypothetical Protein 2745
370	2746	Lipoprotein
371	2747	Hypothetical
372	2748	Hypothetical
373	2749	Hypothetical
374	2750	Hypothetical
375	2751	Hypothetical
376	2752	Hypothetical
377	2753	Protein ClpV1

378	2754	Conserved Hypothetical Protein
379	2755	Rhs Element Vgr Protein
380	2756	Hypothetical
381	2757	Hypothetical
382	2758	Hypothetical
383	2759	Hypothetical
384	2760	Hypothetical
385	2761	Rhs Element Vgr Protein
386	2762	Hypothetical Protein 2762
387	2763	Hypothetical
388	2764	Hypothetical Protein 2764
389	2765	Sulfatase Modifying Factor 1 -Like Protein
390	2766	Hypothetical
391	2767	Hypothetical Protein 2767
392	2768	Hypothetical Protein 2768
393	2769	Hypothetical
394	2770	Hypothetical Protein 2770
395	2771	Hypothetical Protein 2771
396	2772	Hypothetical Protein 2772
397	2773	Hypothetical
398	2774	Conserved Hypothetical Protein
399	2776	Hypothetical
400	2777	Hypothetical
401	2778	Hypothetical
402	2779	Hypothetical
403	2780	Uncharacterized protein Rv0899/MT0922
404	2781	Hypothetical
405	2782	Hypothetical Protein 2782
406	2783	Hypothetical
407	2784	Hypothetical Protein 2784
408	2785	Rhs Element Vgr Protein
409	2787	Hypothetical
410	2788	Hypothetical
411	2789	Hypothetical
412	2791	Rhs Element Vgr Protein
413	2792	Hypothetical
414	2793	Hypothetical Protein 2793
415	2794	Hypothetical

416	2802	Hypothetical Protein 2802
417	2803	6-aminohexanoate-dimer hydrolase
418	2805	Hypothetical
419	2822	Hypothetical
420	2823	6-hydroxy-D-nicotine oxidase
421	2828	Hypothetical Protein 2828
422	2829	Hypothetical
423	2831	Hypothetical Protein 2831
424	2832	ThiJ/PfpI Domain-Containing Protein
425	2833	HTH-type transcriptional repressor nemR
426	2834	Acetamidase/Formamidase
427	2835	Hypothetical Protein 2835
428	2836	NAD-Dependent Epimerase/Dehydratase
429	2843	Cytochrome o ubiquinol oxidase protein CyoD
430	2844	Putative lipoprotein Lxx21020
431	2862	Transposase
432	2864	Hypothetical
433	2867	Hypothetical
434	2869	Uncharacterized sugar epimerase yhfK
435	2884	Putative malate transporter yflS
436	2885	ATP synthase epsilon chain
437	2903	Hypothetical Protein 2903
438	2905	Conserved Hypothetical Protein
439	2907	Hypothetical
440	2908	Tyrosinase
441	2912	Hypothetical
442	2913	Uncharacterized protein YuaQ
443	2914	Hypothetical Protein 2914
444	2915	Hypothetical Protein 2915
445	2916	Hypothetical Protein 2916
446	2917	Hypothetical Protein 2917
447	2918	Hypothetical Protein 2918
448	2920	Putative aldehyde-dehydrogenase-like protein y4uC
449	2923	Hypothetical
450	2924	Hypothetical Protein 2924
451	2925	Hypothetical Protein 2925
452	2926	Hypothetical Protein 2926
453	2927	Putative DNA methyltransferase yeeA

454	2928	Hypothetical Protein 2928
455	2929	Hypothetical
456	2930	Uncharacterized HTH-type transcriptional regulator ycjZ
457	2931	Hypothetical
458	2932	3-oxoacyl-[acyl-carrier-protein] reductase FabG
459	2933	Protein ndvB
460	2934	Hypothetical Protein 2934
461	2935	Hypothetical
462	2936	Hypothetical
463	2937	UPF0173 metal-dependent hydrolase Rxyl_1261
464	2938	Hypothetical Protein 2938
465	2939	Glycine cleavage system transcriptional activator
466	2940	Uncharacterized protein yihR
467	2941	Xylosidase/arabinosidase
468	2942	Beta-glucosidase
469	2943	Xylosidase/arabinosidase
470	2944	Sialate O-Acetylesterase
471	2945	Alpha-glucuronidase
472	2946	Glucose-resistance amylase regulator
473	2949	Xylose isomerase 1
474	2950	Xylulose kinase
475	2951	Uncharacterized protein y4xG
476	2952	Transcription Factor Jumonji Domain-Containing Protein
477	2953	SapC Family Protein
478	2954	NB-Dependent Receptor
479	2955	Catabolite control protein A
480	2957	Hypothetical
481	2960	Uncharacterized transporter YxjC
482	2966	D-(-)-3-hydroxybutyrate oligomer hydrolase
483	2968	Oxidoreductase
484	2969	Hypothetical
485	2970	Lacticin 481/lactococcin biosynthesis protein lcnDR2
486	2971	Hypothetical Protein 2971
487	2973	Hypothetical
488	2981	Hypothetical
489	2989	Ferrichrome-iron receptor
490	2995	Hypothetical Protein 2995
491	3006	Protein CrcB homolog

492	3025	Hypothetical Protein 3025
493	3026	Uncharacterized protein ycaQ
494	3027	Hypothetical Protein 3027
495	3028	Hypothetical Protein 3028
496	3037	Hypothetical Protein 3037
497	3049	Hypothetical Protein 3049
498	3078	Chaperone protein ClpB
499	3079	Radical Activating
500	3081	Hypothetical
501	3111	Hypothetical
502	3116	Peptide chain release factor 2
503	3117	Uncharacterized metalloprotease yggG
504	3122	Hypothetical Protein 3122
505	3137	Signaling protein ykoW
506	3140	Hypothetical
507	3157	Hypothetical
508	3168	Hypothetical Protein 3168
509	3179	Hypothetical
510	3197	Hypothetical Protein 3197
511	3198	Hypothetical Protein 3198
512	3199	Acetyl esterase
513	3200	Hypothetical Protein 3200
514	3201	Hypothetical Protein 3201
515	3202	Hypothetical Protein 3202
516	3205	Uncharacterized 22.5 kDa protein in cps region
517	3206	Hypothetical Protein 3206
518	3207	Putative capsule polysaccharide export protein
519	3208	Lipoprotein
520	3209	Uncharacterized protein yjbG
521	3210	Uncharacterized lipoprotein yjbH
522	3211	Arabinose 5-phosphate isomerase KdsD
523	3212	3-deoxy-D-manno-octulosonate 8-phosphate phosphatase KdsC
524	3213	Hypothetical Protein 3213
525	3214	Hypothetical Protein 3214
526	3215	Capsule Polysaccharide Biosynthesis Protein
527	3216	Hypothetical
528	3217	Putative UDP-glucose 4-epimerase
529	3218	Tyrosine-protein kinase wzc

530	3220	Putative colanic biosynthesis UDP-glucose lipid carrier transferase
531	3229	Ig Family Protein
532	3230	Tail Collar Domain-Containing Protein
533	3232	Tail Collar Domain-Containing Protein
534	3233	GCN5-Like N-Acetyltransferase
535	3234	Hypothetical
536	3238	Hypothetical
537	3246	Monooxygenase
538	3250	Hypothetical Protein 3250
539	3291	Hypothetical Protein 3291
540	3294	Quinate/shikimate dehydrogenase (quinone)
541	3295	Pca regulon regulatory protein
542	3297	3-oxoadipate enol-lactonase 2
543	3298	3-carboxy-cis,cis-muconate cycloisomerase
544	3299	Protocatechuate 3,4-dioxygenase alpha chain
545	3300	Protocatechuate 3,4-dioxygenase beta chain
546	3301	Beta-ketoadipyl-CoA thiolase
547	3302	3-oxoadipate CoA-transferase subunit B
548	3303	3-oxoadipate CoA-transferase subunit A
549	3304	Benzoate transport protein
550	3305	Catabolic 3-dehydroquinate dehydratase
551	3306	Porin B
552	3307	3-dehydroshikimate dehydratase
553	3313	Hypothetical Protein 3313
554	3322	Transposase
555	3340	Hypothetical Protein 3340
556	3346	Hypothetical Protein 3346
557	3352	Acetyltransferase
558	3353	Hypothetical Protein 3353
559	3355	Uncharacterized protein ybeQ
560	3356	ADP-ribosyl-[dinitrogen reductase] glycohydrolase
561	3357	Acetyltransferase
562	3358	Hypothetical
563	3361	Hypothetical Protein 3361
564	3362	Hypothetical Protein 3362
565	3363	Gifsy-1 Prophage Protein
566	3364	Hypothetical
567	3374	Hypothetical Protein 3374

568	3376	Uncharacterized protein yxaH
569	3381	Uncharacterized protein y4iL
570	3384	Hypothetical Protein 3384
571	3388	Hypothetical Protein 3388
572	3389	Hypothetical Protein 3389
573	3416	Hypothetical
574	3436	Uncharacterized protein yvrE
575	3450	Pyridoxal 4-dehydrogenase
576	3452	Hypothetical Protein 3452
577	3454	Blue-light-activated histidine kinase
578	3476	Hypothetical Protein 3476
579	3482	Hypothetical Protein 3482
580	3484	Hypothetical Protein 3484
581	3485	Hypothetical Protein 3485
582	3503	GumN Protein
583	3505	Hypothetical
584	3506	Glutathione S-Transferase
585	3507	Hypothetical Protein 3507
586	3514	Hypothetical Protein 3514
587	3516	Conserved Hypothetical Protein
588	3518	Hypothetical
589	3519	Hypothetical Protein 3519
590	3558	Fatty Acid Desaturase
591	3559	Phosphoesterase PA-Phosphatase
592	3560	Hypothetical
593	3561	Hypothetical
594	3562	Hypothetical
595	3563	Hypothetical
596	3564	Hypothetical Protein 3564
597	3565	Hypothetical
598	3566	Hypothetical
599	3567	Phospholipase D
600	3568	Rhs Element Vgr Protein
601	3570	Hypothetical
602	3585	Hypothetical
603	3586	Hypothetical
604	3587	Hypothetical
605	3588	Hypothetical Protein 3588

606	3589	Rhs Element Vgr Protein
607	3597	Hypothetical Protein 3597
608	3601	Ferric-pseudobactin 358 receptor
609	3616	Hypothetical Protein 3616
610	3617	Hypothetical
611	3618	Hypothetical Protein yijE
612	3619	2OG-Fe(II) Oxygenase
613	3620	Sulfur carrier protein moaD adenylyltransferase
614	3641	Glutathione S-Transferase
615	3652	Hypothetical
616	3653	Hypothetical
617	3663	Hypothetical Protein 3663
618	3667	Hypothetical
619	3684	Transposase
620	3692	Hypothetical
621	3702	Hypothetical
622	3720	MarR Family Transcriptional Regulator
623	3721	Uncharacterized MFS-type transporter ybfB
624	3722	Hypothetical Protein 3722
625	3726	Hypothetical
626	3737	Hypothetical
627	3752	Hypothetical Protein 3752
628	3755	Hypothetical Protein 3755
629	3758	Catalase-peroxidase
630	3759	Glycosyl Hydrolase Family 5 Protein
631	3761	Hypothetical
632	3770	Hypothetical Protein 3770
633	3782	L-lactate permease
634	3784	Sigma factor sigB regulation protein rsbQ
635	3786	Hypothetical Protein 3786
636	3787	Hypothetical
637	3788	Hypothetical
638	3789	Periplasmic trehalase
639	3811	Hypothetical Protein 3811
640	3817	Hypothetical
641	3820	Putative DNA-3-methyladenine glycosylase yfjP
642	3835	Uncharacterized peptidase yuxL
643	3838	Hypothetical

644	3839	Hypothetical
645	3840	Hypothetical
646	3841	Hypothetical
647	3842	Hypothetical
648	3844	Hypothetical Protein 3844
649	3856	Hypothetical
650	3860	Hypothetical Protein 3860
651	3863	Hypothetical
652	3864	NB-Dependent Receptor
653	3869	Hypothetical Protein 3869
654	3874	Acetyltransferase
655	3875	TetR Family Transcriptional Regulator
656	3877	Hypothetical Protein 3877
657	3878	Hypothetical
658	3880	Hypothetical
659	3881	Facilitator Transporter
660	3893	RTX xin-Activating Protein C
661	3894	Iron-regulated protein frpC
662	3895	Leukotoxin secretion protein D
663	3896	Leukotoxin translocation ATP-binding protein LktB
664	3902	Circadian clock protein kinase kaiC
665	3976	Hypothetical
666	3985	Hypothetical
667	3987	Non-heme chloroperoxidase
668	3989	Hypothetical
669	4001	Hypothetical Protein 4001
670	4002	NB-Dependent Siderophore Receptor
671	4005	Hypothetical
672	4006	Hypothetical Protein 4006
673	4007	Hypothetical
674	4008	Putative short-chain type dehydrogenase/reductase Rv0148
675	4009	Hypothetical Protein 4009
676	4011	Conserved Hypothetical Protein
677	4012	Hypothetical Protein 4012
678	4013	Hypothetical Protein 4013
679	4014	UPF0145 protein CV_4322
680	4015	Hypothetical Protein 4015
681	4016	Uncharacterized protein yehP

682	4017	Uncharacterized protein yehM
683	4018	Uncharacterized protein yehL
684	4019	Hypothetical
685	4020	Zinc Finger SWIM Domain-Containing Protein
686	4021	Hypothetical
687	4045	OmpA/MotB Domain Protein
688	4046	Hypothetical Protein 4046
689	4049	Hypothetical
690	4068	Hypothetical Protein 4068
691	4070	Hypothetical
692	4085	Hypothetical Protein 4085
693	4109	Hypothetical Protein 4109
694	4163	Uncharacterized oxidoreductase YurR
695	4164	Hypothetical Protein 4164
696	4165	Hypothetical Protein 4165
697	4176	Hypothetical Protein 4176
698	4199	Hypothetical
699	4203	Hypothetical
700	4228	Hypothetical Protein 4228
701	4250	Uncharacterized protein ynbD
702	4251	Hypothetical Protein 4251
703	4252	Hypothetical Protein 4252
704	4253	Hypothetical Protein 4253
705	4254	Hypothetical Protein 4254
706	4265	Uncharacterized protein yfbP
707	4268	Protein tonB
708	4271	Epimerase family protein yfcH
709	4314	Hypothetical Protein 4314
710	4320	31 kDa outer-membrane immunogenic protein
711	4323	Acyl-CoA dehydrogenase
712	4324	Cell Morphology Protein
713	4325	D-alanine--poly(phosphoribitol) ligase subunit 1
714	4326	Hypothetical Protein 4326
715	4327	Probable poly(beta-D-mannuronate) O-acetylase
716	4328	Alginate biosynthesis protein AlgJ
717	4329	Alginate biosynthesis protein AlgJ
718	4359	Hypothetical
719	4386	Uncharacterized glycosyltransferase slr1943

720	4387	GCN5-Like N-Acetyltransferase
721	4388	Hypothetical
722	4389	Lipopolysaccharide biosynthesis protein rffA
723	4390	Hypothetical Protein 4390
724	4391	Glycosyl Transferase Family Protein
725	4392	Glycosyl Transferase Family Protein
726	4410	Hypothetical
727	4439	Hypothetical Protein 4439
728	4449	Phospholipase/Carboxylesterase
729	4467	Hypothetical
730	4479	Uncharacterized protein yafM
731	4480	Hypothetical Protein 4480
732	4481	Hypothetical Protein 4481
733	4485	Hypothetical Protein 4485
734	4486	CopY Family Transcriptional Regulator
735	4487	Peptidase
736	4489	Hypothetical Protein 4489
737	4490	Hypothetical Protein 4490
738	4491	Hypothetical Protein 4491
739	4492	Hypothetical Protein 4492
740	4493	Hypothetical Protein 4493
741	4495	Hypothetical Protein 4495
742	4496	Hypothetical
743	4497	Paar Motif Family Protein
744	4498	Hypothetical
745	4499	Hypothetical
746	4500	Hypothetical
747	4501	Hypothetical
748	4502	Hypothetical
749	4503	Hypothetical Protein 4503
750	4504	Hypothetical
751	4505	Hypothetical Protein 4505
752	4506	Hypothetical Protein 4506
753	4507	Transcriptional Factor
754	4508	Hypothetical Protein 4508
755	4509	Hypothetical
756	4510	Hypothetical
757	4511	Hypothetical Protein 4511

758	4512	Hypothetical Protein 4512
759	4514	Hypothetical Protein 4514
760	4516	Hypothetical
761	4517	Hypothetical
762	4521	Hypothetical Protein 4521
763	4531	Hypothetical Protein 4531
764	4537	Hypothetical Protein 4537
765	4544	Hypothetical Protein 4544
766	4564	Hypothetical
767	4565	Hypothetical
768	4574	Hypothetical Protein 4574
769	4578	Hypothetical
770	4594	Hypothetical Protein 4594
771	4603	Uncharacterized protein NMB0459
772	4623	PfkB Domain Protein
773	4625	Uncharacterized HTH-type transcriptional regulator ycfQ
774	4626	Xylose Isomerase Domain-Containing Protein
775	4627	Soluble epoxide hydrolase
776	4628	Lipase 2
777	4633	Hypothetical Protein 4633
778	4634	Hypothetical
779	4635	Hypothetical Protein 4635
780	4636	Hypothetical
781	4637	Hypothetical
782	4638	Protein rhsD
783	4639	Hypothetical Protein 4639
784	4640	Hypothetical
785	4641	Type VI Secretion System Vgr Family Protein
786	4653	Hypothetical
787	4667	Hypothetical Protein 4667
788	4685	Hypothetical Protein 4685
789	4687	Hypothetical Protein 4687
790	4696	Hypothetical
791	4706	Hypothetical
792	4707	RNA polymerase sigma factor sigK
793	4713	Plasmid Maintenance System Killer
794	4714	Virulence-associated protein I
795	4715	5-methyltetrahydropteroyltriglutamate-- homocysteine methyltransferase

796	4719	Hypothetical
797	4725	Hypothetical
798	4726	Hypothetical
799	4729	Hypothetical
800	4742	Hypothetical Protein 4742
801	4745	Rhs Element Vgr Protein
802	4746	Hypothetical
803	4747	Hypothetical
804	4752	Hypothetical
805	4775	Hypothetical Protein 4775
806	4790	Phospholipase/Carboxylesterase
807	4791	Nodulation protein nolG
808	4792	Multidrug resistance protein mdtA
809	4793	Tetracycline repressor protein class A from transposon 1721
810	4794	Hypothetical
811	4795	Blue light- and temperature-regulated antirepressor YcgF
812	4796	Hypothetical Protein 4796
813	4797	Beta-Lactamase Domain-Containing Protein
814	4798	Hypothetical
815	4799	Hypothetical
816	4801	Hypothetical
817	4802	Hypothetical
818	4803	Hypothetical
819	4804	Hypothetical
820	4805	Hypothetical Protein 4805
821	4806	Uncharacterized protein HI_0912
822	4809	Capsular synthesis regulator component B
823	4828	Hypothetical Protein 4828
824	4829	Hypothetical
825	4830	Hypothetical Protein 4830
826	4832	Thioredoxin reductase
827	4837	Hypothetical Protein 4837
828	4851	Uncharacterized transporter MTH_1382
829	4854	Transcriptional Regulator
830	4866	Hypothetical Protein 4866
831	4867	Putative membrane protein igaA homolog
832	4876	Hypothetical Protein 4876
833	4880	Hypothetical

834	4883	Hypothetical Protein 4883
835	4888	Hypothetical
836	4891	Trypsin
837	4899	Probable serine/threonine-protein kinase Sps1
838	4900	ECF Subfamily RNA Polymerase Sigma-24 Factor
839	4902	Hypothetical
840	4912	Hypothetical
841	4913	Hypothetical
842	4914	Hypothetical Protein 4914
843	4915	Rhs Element Vgr Protein
844	4918	Hypothetical
845	4919	Probable rhamnogalacturonan acetylesterase yesY
846	4920	2-dehydro-3-deoxy-D-gluconate 5-dehydrogenase
847	4921	4-deoxy-L-threo-5-hexosulose-uronate ketol-isomerase
848	4923	Putative beta-xylosidase
849	4924	Pectate Lyase
850	4925	Uncharacterized protein y4mL
851	4926	C4-Dicarboxylate Transport Small Permease Component
852	4927	Uncharacterized protein yiiZ
853	4928	2-Keto-4-Pentenoate Hydratase
854	4929	Endo-1,4-beta-xylanase B
855	4930	Para-nitrobenzyl esterase
856	4931	Probable rhamnogalacturonan acetylesterase yesY
857	4933	Uncharacterized isomerase PM1503
858	4934	Hypothetical
859	4935	NB-Dependent Receptor
860	4936	NB-Dependent Receptor
861	4939	Cupin 2 Domain-Containing Protein
862	4940	Acetoin catabolism regulatory protein
863	4941	Acetaldehyde dehydrogenase 2
864	4942	Alcohol dehydrogenase
865	4943	Hypothetical
866	4953	Hypothetical Protein 4953
867	4954	Putative ABC transporter ATP-binding protein MK0182
868	4956	Hypothetical Protein 4956
869	4957	Hypothetical Protein 4957
870	4958	Macrolide export ATP-binding/permease protein MacB
871	4959	Uncharacterized ABC transporter ATP-binding protein YknY

872	4960	Macrolide-specific efflux protein macA
873	4961	Hypothetical
874	4962	Transposase
875	4963	Hypothetical Protein 4963
876	4964	Hypothetical Protein 4964
877	4978	Hypothetical Protein 4978
878	4986	Hypothetical
879	4988	Hypothetical
880	4996	Uncharacterized oxidoreductase YccK
881	4997	Uncharacterized HTH-type transcriptional regulator ycaN
882	4998	Hypothetical Protein 4998
883	5002	Oxygen-insensitive NAD(P)H nitroreductase
884	5016	Hypothetical
