# Supplementary material for: Stenotrophomonas comparative genomics reveals genes and functions that differentiate beneficial and pathogenic bacteria
Source: BMC Genomics. 2014 Jun 18;15(1):482. doi: 10.1186/1471-2164-15-482 (PMC4101175; doi:10.1186/1471-2164-15-482)
Supplement: Supplementary file 3 — Additional file 3: Table S2: The list of the 1230 S. maltophilia K279a specific genes that are absent from S. rhizophila DSM14405T. (PDF 64 KB) [file 12864_2013_6236_MOESM3_ESM.pdf]

Supplementary Table2: The list of the 1230 S. maltophilia K279a specific genes that are absent from S. rhizophila DSM14405T

Nr.	locus tag	(putative) product
1	Smlt0003	hypothetical protein
2	Smlt0017	putative DNA-binding protein
3	Smlt0018	hypothetical protein
4	Smlt0020	hypothetical protein
5	Smlt0021	hypothetical protein
6	Smlt0023	putative integrase/recombinase
7	Smlt0024	hypothetical protein
8	Smlt0025	hypothetical protein
9	Smlt0027	putative ISXac3 related insertion element
10	Smlt0028	hypothetical protein
11	Smlt0029	hypothetical protein
12	Smlt0032	putative MFS family transporter protein
13	Smlt0033	hypothetical protein
14	Smlt0034	putative transmembrane anchor protein
15	Smlt0035	putative transmembrane protein
16	Smlt0036	putative AcrA/AcrD/AcrF family protein
17	Smlt0037	putative cation efflux component protein
18	Smlt0038	putative cation efflux transporter component
19	Smlt0039	putative transmembrane protein
20	Smlt0040	hypothetical protein
21	Smlt0042	putative RHS-repeat-containing protein
22	Smlt0043	putative wall-associated protein
23	Smlt0044	putative RHS-repeat-containing protein
24	Smlt0045	hypothetical protein
25	Smlt0056	putative integrase
26	Smlt0057	putative phage DNA-binding protein
27	Smlt0058	hypothetical protein
28	Smlt0059	putative bacteriophage transcriptional regulator protein
29	Smlt0060	hypothetical protein
30	Smlt0061	hypothetical protein
31	Smlt0062	hypothetical protein
32	Smlt0063	putative primase domain-containing protein
33	Smlt0064	hypothetical protein
34	Smlt0065	hypothetical protein
35	Smlt0066	putative phage portal vertex protein

36	Smlt0067	hypothetical protein
37	Smlt0068	hypothetical protein
38	Smlt0069	hypothetical protein
39	Smlt0076	hypothetical protein
40	Smlt0079	hypothetical protein
41	Smlt0107	putative sensor histidine kinase transcriptional regulator two-component regulator
42	Smlt0113	hypothetical protein
43	Smlt0114	hypothetical protein
44	Smlt0135	putative HTH-type transcriptional regulator HipB
45	Smlt0148	hypothetical protein
46	Smlt0162	hypothetical protein
47	Smlt0173	hypothetical protein
48	Smlt0175	hypothetical protein
49	Smlt0176	putative arsenite transmembrane efflux pump transporter protein
50	Smlt0177	putative arsenate reductase ArsC
51	Smlt0178	putative ArsR family transcriptional regulator
52	Smlt0179	putative arsenic resistance ArsH-like protein
53	Smlt0180	putative transmembrane protein
54	Smlt0183	hypothetical protein
55	Smlt0187	putative peroxidase
56	Smlt0193	hypothetical protein
57	Smlt0194	hypothetical protein
58	Smlt0214	hypothetical protein
59	Smlt0215	putative hexapeptide transferase
60	Smlt0216	putative transmembrane glycosyl transferase
61	Smlt0217	putative N-acetyl glucosamine deacetylase
62	Smlt0218	putative o-methyltransferase
63	Smlt0249	hypothetical protein
64	Smlt0259	hypothetical protein
65	Smlt0271	hypothetical protein
66	Smlt0272	hypothetical protein
67	Smlt0273	putative peptidoglycan-binding motif protein
68	Smlt0279	hypothetical protein
69	Smlt0285	putative phage integrase
70	Smlt0286	hypothetical protein
71	Smlt0287	hypothetical protein
72	Smlt0288	hypothetical protein
73	Smlt0289	hypothetical protein

74	Smlt0290	putative phage-like protein
75	Smlt0291	hypothetical protein
76	Smlt0293	hypothetical protein
77	Smlt0294	hypothetical protein
78	Smlt0295	hypothetical protein
79	Smlt0296	putative phage-like protein
80	Smlt0297	hypothetical protein
81	Smlt0298	hypothetical protein
82	Smlt0299	putative phage-like protein
83	Smlt0300	putative phage tail protein
84	Smlt0301	putative phage tail protein
85	Smlt0302	hypothetical protein
86	Smlt0303	putative phage tail protein
87	Smlt0304	putative major tail tube protein
88	Smlt0305	putative major tail sheath protein
89	Smlt0306	putative baseplate assembly protein W
90	Smlt0307	putative baseplate assembly protein V
91	Smlt0308	hypothetical protein
92	Smlt0310	putative tail protein I
93	Smlt0311	putative baseplate assembly protein J
94	Smlt0312	putative tail completion protein S
95	Smlt0313	putative tail completion protein R
96	Smlt0314	hypothetical protein
97	Smlt0315	putative phage lytic enzyme
98	Smlt0316	hypothetical protein
99	Smlt0317	hypothetical protein
100	Smlt0318	putative tail protein X
101	Smlt0319	putative head completion/stabilization protein
102	Smlt0320	putative terminase, endonuclease subunit (gpm)
103	Smlt0321	putative minor capsid protein h1/minor capsid protein h2/major capsid protein n*
104	Smlt0322	putative capsid scaffolding protein
105	Smlt0323	putative phage terminase, ATPase subunit (gpp)
106	Smlt0324	putative presumed portal vertex protein (gpq)
107	Smlt0325	putative site-specific DNA-methyltransferase
108	Smlt0327	hypothetical protein
109	Smlt0328	hypothetical protein
110	Smlt0329	hypothetical protein
111	Smlt0330	putative DNA recombinase

112	Smlt0331	hypothetical protein
113	Smlt0332	hypothetical protein
114	Smlt0333	hypothetical protein
115	Smlt0336	hypothetical protein
116	Smlt0337	hypothetical protein
117	Smlt0340	putative ankyrin repeat-containing protein
118	Smlt0349	putative transmembrane protein
119	Smlt0351	putative transmembrane protein
120	Smlt0352	putative ATP-binding component of ABC transporter
121	Smlt0354	hypothetical protein
122	Smlt0355	hypothetical protein
123	Smlt0368	putative transposase
124	Smlt0369	putative transposase
125	Smlt0370	hypothetical protein
126	Smlt0382	hypothetical protein
127	Smlt0389	putative two-component LuxR family transcriptional regulator
128	Smlt0400	putative two-component response regulator transcriptional regulator
129	Smlt0427	putative transmembrane protein
130	Smlt0430	putative transmembrane protein
131	Smlt0465	putative transposase for IS element
132	Smlt0480	hypothetical protein
133	Smlt0494	putative ISXac3 like transposase
134	Smlt0497	hypothetical protein
135	Smlt0498	hypothetical protein
136	Smlt0498A	putative protease
137	Smlt0500	hypothetical protein
138	Smlt0501	hypothetical protein
139	Smlt0502	hypothetical protein
140	Smlt0504	hypothetical protein
141	Smlt0505	hypothetical protein
142	Smlt0506	hypothetical protein
143	Smlt0509	putative transmembrane protein
144	Smlt0510	putative transmembrane protein
145	Smlt0510A	hypothetical protein
146	Smlt0510B	hypothetical protein
147	Smlt0514	hypothetical protein
148	Smlt0517	putative transmembrane transporter
149	Smlt0518	putative transposase

150	Smlt0534	hypothetical protein
151	Smlt0537	hypothetical protein
152	Smlt0538	putative transmembrane anchor protein
153	Smlt0539	hypothetical protein
154	Smlt0540	hypothetical protein
155	Smlt0558	putative transposase-like protein
156	Smlt0583	hypothetical protein
157	Smlt0596	putative two component sensor histidine kinase transcriptional regulator
158	Smlt0597	putative two-component response regulator transcriptional regulator
159	Smlt0598	putative O-antigen biosynthesis aminotransferase
160	Smlt0599	hypothetical protein
161	Smlt0622	putative glycosyl transferase
162	Smlt0623	putative glycosyltransferase, fusion protein
163	Smlt0624	putative transmembrane protein
164	Smlt0625	putative lipase
165	Smlt0626	putative transmembrane protein
166	Smlt0627	putative transmembrane GtrA-like cell surface polysaccharide biosynthesis protein
167	Smlt0628	putative transmembrane cell surface polysaccharide biosynthesis protein
168	Smlt0629	hypothetical protein
169	Smlt0630	putative transmembrane anchor NAD-dependent epimerase/dehydratase/dehydrogenase
170	Smlt0631	hypothetical protein
171	Smlt0632	short chain dehydrogenase
172	Smlt0633	putative FAD-binding oxidoreductase
173	Smlt0634	hypothetical protein
174	Smlt0635	putative transmembrane protein
175	Smlt0636	putative glycosyl transferase
176	Smlt0637	putative transmembrane protein
177	Smlt0638	putative xylose isomerase
178	Smlt0639	hypothetical protein
179	Smlt0640	hypothetical protein
180	Smlt0641	putative undecaprenyl-phosphate 4-deoxy-4-formamido-l-arabinose transferase
181	Smlt0642	putative transmembrane protein
182	Smlt0644	putative transmembrane sulfatase
183	Smlt0651	putative transmembrane protein
184	Smlt0654	putative helicase
185	Smlt0657	putative glycosyltransferase
186	Smlt0678	hypothetical protein
187	Smlt0700	hypothetical protein

188	Smlt0704	hypothetical protein
189	Smlt0706	putative fimbrial adhesin protein
190	Smlt0707	putative pili chaperone protein
191	Smlt0708	putative outer membrane usher protein mrkc precursor
192	Smlt0709	putative fimbria adhesin protein
193	Smlt0721	putative transmembrane protein
194	Smlt0724	putative transmembrane protein
195	Smlt0737	putative O-antigen acetylase
196	Smlt0768	putative alpha/beta hydrolase fold
197	Smlt0781	putative acyltransferase
198	Smlt0782	hypothetical protein
199	Smlt0788	putative transmembrane protein
200	Smlt0789	putative LysR family transcriptional regulator
201	Smlt0790	putative transporter
202	Smlt0806	putative transmembrane MFS family protein
203	Smlt0830	hypothetical protein
204	Smlt0831	putative transmembrane protein
205	Smlt0945	putative ribosomal large subunit pseudouridine synthase A
206	Smlt0985	hypothetical protein
207	Smlt0987	hypothetical protein
208	Smlt1001	putative autotransporter
209	Smlt1009	putative glycine-rich autotransporter protein
210	Smlt1011	putative transmembrane protein
211	Smlt1012	hypothetical protein
212	Smlt1013	putative restriction-modification system methyltransferase
213	Smlt1038	putative tautomerase
214	Smlt1039	hypothetical protein
215	Smlt1040	putative phage tail protein
216	Smlt1041	putative phage tail protein
217	Smlt1042	putative bacteriophage tail protein
218	Smlt1043	hypothetical protein
219	Smlt1044	putative major tail tube protein
220	Smlt1045	putative bacteriophage major tail sheath protein
221	Smlt1046	hypothetical protein
222	Smlt1047	putative phage tail protein
223	Smlt1048	putative bacteriophage tail protein I
224	Smlt1049	putative phage baseplate assembly protein
225	Smlt1050	putative phage baseplate assembly protein

226	Smlt1051	putative baseplate assembly protein
227	Smlt1052	putative phage-like protein
228	Smlt1053	hypothetical protein
229	Smlt1054	putative endolysin/phage lysozyme
230	Smlt1055	hypothetical protein
231	Smlt1056	hypothetical protein
232	Smlt1057	hypothetical protein
233	Smlt1058	putative phage-like protein
234	Smlt1059	hypothetical protein
235	Smlt1060	putative regulatory protein
236	Smlt1061	putative phage integrase
237	Smlt1064	hypothetical protein
238	Smlt1065	putative phage-like protein
239	Smlt1067	putative TonB-dependent receptor
240	Smlt1068	putative transmembrane protein
241	Smlt1071	putative fluoroquinolone resistance protein
242	Smlt1073	putative glutathione S-transferase
243	Smlt1074	putative LysR family regulatory protein
244	Smlt1134	putative DNA transport competence protein
245	Smlt1147	hypothetical protein
246	Smlt1156	hypothetical protein
247	Smlt1167	putative phenazine biosynthesis-like protein
248	Smlt1182	putative transmembrane protein
249	Smlt1183	hypothetical protein
250	Smlt1201	putative thioesterase
251	Smlt1202	putative transmembrane protein
252	Smlt1206	hypothetical protein
253	Smlt1207	putative transmembrane protein
254	Smlt1208	putative DNA-binding protein
255	Smlt1209	putative transmembrane transporter domain-containing protein
256	Smlt1211	putative transmembrane protein
257	Smlt1212	hypothetical protein
258	Smlt1213	hypothetical protein
259	Smlt1214	hypothetical protein
260	Smlt1215	putative transmembrane DoxX family protein
261	Smlt1216	hypothetical protein
262	Smlt1217	putative exported thioredoxin
263	Smlt1218	putative two-component response regulator transcriptional regulator

264	Smlt1219	putative transmembane sensor histidine kinase transcriptional regulator
265	Smlt1220	hypothetical protein
266	Smlt1222	putative inosine-uridine preferring nucleoside hydrolase
267	Smlt1223	hypothetical protein
268	Smlt1236	hypothetical protein
269	Smlt1243	hypothetical protein
270	Smlt1282	hypothetical protein
271	Smlt1283	putative conjugal transfer protein
272	Smlt1284	putative conjugal transfer protein
273	Smlt1285	conjugal transfer protein TrbF
274	Smlt1286	conjugal transfer protein TrbL
275	Smlt1287	conjugal transfer protein TrbJ
276	Smlt1288	conjugal transfer ATPase TrbE
277	Smlt1289	putative conjugal transfer protein
278	Smlt1290	putative conjugal transfer protein
279	Smlt1291	putative conjugal transfer protein
280	Smlt1292	hypothetical protein
281	Smlt1293	conjugal transfer coupling protein TraG
282	Smlt1294	hypothetical protein
283	Smlt1298	hypothetical protein
284	Smlt1299	putative NAD(P)H dehydrogenase
285	Smlt1300	putative LysR family transcriptional regulator
286	Smlt1301	hypothetical protein
287	Smlt1303	hypothetical protein
288	Smlt1305	putative MerR family transcriptional regulator
289	Smlt1308	hypothetical protein
290	Smlt1309	hypothetical protein
291	Smlt1310	putative transmembrane anchor conjugal transfer protein
292	Smlt1311	hypothetical protein
293	Smlt1312	putative parB partition protein
294	Smlt1313	putative ParA/CobQ/CobB/MinD nucleotide binding domain-containing protein
295	Smlt1314	putative RepA-like replication protein
296	Smlt1315	hypothetical protein
297	Smlt1316	hypothetical protein
298	Smlt1318	hypothetical protein
299	Smlt1319	putative HTH transcriptional regulator
300	Smlt1320	hypothetical protein
301	Smlt1320A	hypothetical protein

302	Smlt1322	hypothetical protein
303	Smlt1323	putative transposase
304	Smlt1324	hypothetical protein
305	Smlt1325	hypothetical protein
306	Smlt1326	putative ParB-like nuclease domain-containing protein
307	Smlt1327	hypothetical protein
308	Smlt1328	hypothetical protein
309	Smlt1329	hypothetical protein
310	Smlt1331	putative transmembrane protein
311	Smlt1333	hypothetical protein
312	Smlt1334	hypothetical protein
313	Smlt1335	hypothetical protein
314	Smlt1336	putative prophage integrase
315	Smlt1345	hypothetical protein
316	Smlt1349	putative RNA polymerase sigma factor
317	Smlt1350	putative outer membrane autotransporter
318	Smlt1353	hypothetical protein
319	Smlt1354	hypothetical protein
320	Smlt1355	hypothetical protein
321	Smlt1356	hypothetical protein
322	Smlt1357	hypothetical protein
323	Smlt1358	putative transmembrane protein
324	Smlt1359	hypothetical protein
325	Smlt1385	putative catalase
326	Smlt1386	hypothetical protein
327	Smlt1389	putative activation/secretion
328	Smlt1390	putative outer membrane surface heamagglutinin protein
329	Smlt1396A	hypothetical protein
330	Smlt1399	hypothetical protein
331	Smlt1400	putative transmembrane protein
332	Smlt1401	hypothetical protein
333	Smlt1408	chromosome replication initiation inhibitor protein
334	Smlt1409	putative transmembrane LysE family transport protein
335	Smlt1413	hypothetical protein
336	Smlt1414	hypothetical protein
337	Smlt1415	putative diaminobutyrate--2-oxoglutarate aminotransferase
338	Smlt1416	putative phosphatase
339	Smlt1417	putative nucleotide sugar transaminase

340	Smlt1418	putative transmembrane protein
341	Smlt1419	putative transmembrane protein
342	Smlt1420	hypothetical protein
343	Smlt1421	putative two-component system sensor histidine kinase
344	Smlt1422	putative two-component response regulator transcriptional regulator
345	Smlt1426	outer membrane receptor FepA
346	Smlt1427	hypothetical protein
347	Smlt1429	putative endonuclease V
348	Smlt1450	hypothetical protein
349	Smlt1453	hypothetical protein
350	Smlt1456	hypothetical protein
351	Smlt1466	hypothetical protein
352	Smlt1472	glucose-1-phosphatase/inositol phosphatase
353	Smlt1473	poly(beta-D-mannuronate) lyase
354	Smlt1509	hypothetical protein
355	Smlt1530A	putative transmembrane protein
356	Smlt1532	putative LysR family regulatory protein
357	Smlt1533	putative transmembrane protein
358	Smlt1572	putative transmembrane protein
359	Smlt1576	putative transmembrane protein
360	Smlt1582	hypothetical protein
361	Smlt1593	putative transmembrane protein
362	Smlt1594	hypothetical protein
363	Smlt1601	putative glutathione S-transferase
364	Smlt1615	putative Alkylhydroperoxidase/carboxymuconolactone deacetylase
365	Smlt1629	hypothetical protein
366	Smlt1630	putative transmembrane protein
367	Smlt1631	putative redoxin
368	Smlt1633	hypothetical protein
369	Smlt1651	putative outer membrane efflux protein
370	Smlt1652	putative ABC transporter membrane protein
371	Smlt1653	putative ABC transporter ATP-binding protein
372	Smlt1654	putative HlyD family secretion protein
373	Smlt1655	putative transmembrane protein
374	Smlt1656	putative transmembrane protein
375	Smlt1657	hypothetical protein
376	Smlt1658	hypothetical protein
377	Smlt1659	hypothetical protein

378	Smlt1660	putative modification methylase
379	Smlt1661	hypothetical protein
380	Smlt1663	putative ISXac3 like transposase
381	Smlt1664	putative transmembrane protein
382	Smlt1665	hypothetical protein
383	Smlt1666	hypothetical protein
384	Smlt1668	putative acetyltransferase
385	Smlt1669	putative aminoglycoside 2'-N-acetyltransferase
386	Smlt1671	hypothetical protein
387	Smlt1672	putative ArsR family transcriptional regulator
388	Smlt1675	hypothetical protein
389	Smlt1677	hypothetical protein
390	Smlt1680	hypothetical protein
391	Smlt1681	hypothetical protein
392	Smlt1683	putative transmembrane camphor resistance-like (CrcB-like) protein
393	Smlt1684	hypothetical protein
394	Smlt1685	hypothetical protein
395	Smlt1707	putative carboxypeptidase
396	Smlt1710	hypothetical protein
397	Smlt1713	hypothetical protein
398	Smlt1714	hypothetical protein
399	Smlt1725	hypothetical protein
400	Smlt1731	hypothetical protein
401	Smlt1746	putative TonB-dependent receptor protein
402	Smlt1749	hypothetical protein
403	Smlt1753	putative TonB dependent receptor protein
404	Smlt1754	putative alkaline phosphatase
405	Smlt1757	putative cytochrome c family protein
406	Smlt1758	putative alcohol dehydrogenase cytochrome c subunit
407	Smlt1761	hypothetical protein
408	Smlt1781	putative transmembrane protein
409	Smlt1817	putative ArsR family transcriptional regulator
410	Smlt1818	putative Hsp90 family heat shock chaperone protein
411	Smlt1838	putative transmembrane protein
412	Smlt1839	putative LuxR family transcriptional regulator
413	Smlt1840	chaperone protein HchA
414	Smlt1841	hypothetical protein
415	Smlt1842	hypothetical protein

416	Smlt1843	hypothetical protein
417	Smlt1844	putative modification methylase
418	Smlt1844A	putative modification methylase
419	Smlt1844B	hypothetical protein
420	Smlt1845	putative ISXac3 like transposase
421	Smlt1846A	hypothetical protein
422	Smlt1846B	hypothetical protein
423	Smlt1849	putative transmembrane protein
424	Smlt1849A	hypothetical protein
425	Smlt1850	hypothetical protein
426	Smlt1851	putative glycosidase
427	Smlt1852	putative transmembrane protein
428	Smlt1853	hypothetical protein
429	Smlt1854	tail assembly protein
430	Smlt1855	hypothetical protein
431	Smlt1856	putative transmembrane protein
432	Smlt1857	putative phage tail assembly protein
433	Smlt1858	hypothetical protein
434	Smlt1859	hypothetical protein
435	Smlt1860	putative tail length phage protein
436	Smlt1861	hypothetical protein
437	Smlt1862	hypothetical protein
438	Smlt1863	hypothetical protein
439	Smlt1864	hypothetical protein
440	Smlt1865	hypothetical protein
441	Smlt1866	putative phage-like protein
442	Smlt1867	hypothetical protein
443	Smlt1868	putative head-tail preconnector protein
444	Smlt1869	putative portal protein B
445	Smlt1870	hypothetical protein
446	Smlt1871	hypothetical protein
447	Smlt1872	hypothetical protein
448	Smlt1873	putative phage terminase large subunit
449	Smlt1874	hypothetical protein
450	Smlt1875	hypothetical protein
451	Smlt1876	hypothetical protein
452	Smlt1877	hypothetical protein
453	Smlt1878	putative transposition helper protein

454	Smlt1879	putative transposase
455	Smlt1880	hypothetical protein
456	Smlt1881	putative DNA methyltransferase
457	Smlt1882	putative DNA methylase
458	Smlt1883	hypothetical protein
459	Smlt1884	hypothetical protein
460	Smlt1885	hypothetical protein
461	Smlt1886	hypothetical protein
462	Smlt1887	hypothetical protein
463	Smlt1888	hypothetical protein
464	Smlt1889	hypothetical protein
465	Smlt1890	hypothetical protein
466	Smlt1891	hypothetical protein
467	Smlt1892	putative phage-like protein
468	Smlt1893	hypothetical protein
469	Smlt1894	hypothetical protein
470	Smlt1895	hypothetical protein
471	Smlt1896	hypothetical protein
472	Smlt1897	hypothetical protein
473	Smlt1898	putative HTH transcriptional regulator
474	Smlt1900	hypothetical protein
475	Smlt1901	putative recombinase/resolvase
476	Smlt1902	putative phage-like protein
477	Smlt1903	putative LacI family transcriptional regulator
478	Smlt1905	hypothetical protein
479	Smlt1906	putative phage-like protein
480	Smlt1907	putative phage integrase
481	Smlt1908	putative phage excisionase
482	Smlt1911	hypothetical protein
483	Smlt1912	hypothetical protein
484	Smlt1913	hypothetical protein
485	Smlt1914	hypothetical protein
486	Smlt1915	hypothetical protein
487	Smlt1916	hypothetical protein
488	Smlt1917	hypothetical protein
489	Smlt1918	recombination associated protein
490	Smlt1920	hypothetical protein
491	Smlt1921	hypothetical protein

492	Smlt1922	putative transcriptional regulator
493	Smlt1923	hypothetical protein
494	Smlt1924	hypothetical protein
495	Smlt1925	hypothetical protein
496	Smlt1926	hypothetical protein
497	Smlt1927	hypothetical protein
498	Smlt1929	hypothetical protein
499	Smlt1930	hypothetical protein
500	Smlt1931	hypothetical protein
501	Smlt1932	hypothetical protein
502	Smlt1933	hypothetical protein
503	Smlt1934	hypothetical protein
504	Smlt1935	hypothetical protein
505	Smlt1936	hypothetical protein
506	Smlt1937	hypothetical protein
507	Smlt1938	hypothetical protein
508	Smlt1939	hypothetical protein
509	Smlt1940	putative transmembrane protein
510	Smlt1941	putative transmembrane protein
511	Smlt1944	putative transmembrane phage lysozyme
512	Smlt1946	hypothetical protein
513	Smlt1947	hypothetical protein
514	Smlt1948	hypothetical protein
515	Smlt1949	hypothetical protein
516	Smlt1950	hypothetical protein
517	Smlt1951	hypothetical protein
518	Smlt1952	putative phage tail fiber protein
519	Smlt1953	putative phage terminase
520	Smlt1954	putative DNA packaging protein gp17
521	Smlt1955	hypothetical protein
522	Smlt1956	hypothetical protein
523	Smlt1957	hypothetical protein
524	Smlt1958	hypothetical protein
525	Smlt1959	putative phage-like protein
526	Smlt1960	hypothetical protein
527	Smlt1961	putative phage-like protein
528	Smlt1962	hypothetical protein
529	Smlt1963	putative phage-like protein

530	Smlt1964	hypothetical protein
531	Smlt1965	hypothetical protein
532	Smlt1967	hypothetical protein
533	Smlt1968	hypothetical protein
534	Smlt1969	hypothetical protein
535	Smlt1970	hypothetical protein
536	Smlt1971	hypothetical protein
537	Smlt1972	putative phage-like protein
538	Smlt1973	hypothetical protein
539	Smlt1974	hypothetical protein
540	Smlt1975	hypothetical protein
541	Smlt1976	hypothetical protein
542	Smlt1978	hypothetical protein
543	Smlt1979	hypothetical protein
544	Smlt1980	hypothetical protein
545	Smlt1982	putative transmembrane protein
546	Smlt2061	putative LysE family transmembrane transporter
547	Smlt2068	putative repeat-containing protein
548	Smlt2074	hypothetical protein
549	Smlt2075	hypothetical protein
550	Smlt2076	hypothetical protein
551	Smlt2077A	hypothetical protein
552	Smlt2079	hypothetical protein
553	Smlt2082	putative transmembrane anchor protein
554	Smlt2083	putative transmembrane protein
555	Smlt2085	putative transmembrane protein
556	Smlt2086	putative hydrolase
557	Smlt2088	putative transmembrane protein
558	Smlt2089	hypothetical protein
559	Smlt2090	putative ArsR family regulatory protein
560	Smlt2091	putative Hsp90 ATPase like protein
561	Smlt2092	putative acetyltransferase
562	Smlt2094	putative TetR family transcriptional regulator
563	Smlt2095	putative transmembrane protein
564	Smlt2096	putative chemotaxis protein CheW
565	Smlt2097	putative FAD containing sensors of blue-light domain-containing protein
566	Smlt2109	hypothetical protein
567	Smlt2112	putative TetR family transcriptional regulator

568	Smlt2113	putative transmembrane EAL domain signalling protein
569	Smlt2114	putative LysR family transcriptional activator
570	Smlt2115	putative major facilitator superfamily protein
571	Smlt2116	putative dehydrogenase/reductase
572	Smlt2118	putative ArsR family transcriptional regulator
573	Smlt2119	putative transmembrane protein
574	Smlt2121	putative DoxX family transmembrane protein
575	Smlt2123	putative TetR family transcriptional regulator
576	Smlt2124	putative transmembrane protein
577	Smlt2134	putative acetyltransferase
578	Smlt2135	putative acetyltransferase
579	Smlt2136	putative AraC family regulatory protein
580	Smlt2137	putative universal stress family protein
581	Smlt2138	putative HTH-type transcriptional regulator
582	Smlt2139	putative two-component system response regulator
583	Smlt2142	putative sensor kinase / response regulator fusion protein
584	Smlt2143	putative CheB methylesterase
585	Smlt2144	putative two component system histidine kinase/response regulator fusion protein
586	Smlt2144A	hypothetical protein
587	Smlt2148	putative ArsR family regulatory protein
588	Smlt2149	putative transmembrane protein
589	Smlt2150	putative transmembrane protein
590	Smlt2171	hypothetical protein
591	Smlt2190	hypothetical protein
592	Smlt2191	putative transmembrane protein
593	Smlt2213	hypothetical protein
594	Smlt2216	3-ketoacyl-(acyl-carrier-protein) reductase
595	Smlt2229	hypothetical protein
596	Smlt2230	putative oxidoreductase/aldehyde-dehydrogenase
597	Smlt2239	putative TetR family regulatory protein
598	Smlt2240	putative high-affinity choline transport protein
599	Smlt2242	putative small protein A homolog
600	Smlt2246	hypothetical protein
601	Smlt2247	hypothetical protein
602	Smlt2275	putative esterase/peptidase
603	Smlt2294	hypothetical protein
604	Smlt2328	putative transposase
605	Smlt2329	hypothetical protein

606	Smlt2350	hypothetical protein
607	Smlt2351	putative transposase
608	Smlt2352	hypothetical protein
609	Smlt2360	putative transposase
610	Smlt2379	hypothetical protein
611	Smlt2381	putative two-component response regulator protein
612	Smlt2382	putative two-component sensor histidine kinase
613	Smlt2384	putative transmembrane TspO/MBR tryptophan-rich family protein
614	Smlt2386	hypothetical protein
615	Smlt2387	putative transmembrane protein
616	Smlt2388	putative transmembrane protein
617	Smlt2389	hypothetical protein
618	Smlt2390	putative transmembrane protein
619	Smlt2391	putative transmembrane protein
620	Smlt2392	putative replication protein
621	Smlt2393	putative phage DNA binding (helix destabilizing) protein
622	Smlt2394	putative transmembrane protein
623	Smlt2395	putative transmembrane protein
624	Smlt2397	putative transmembrane phage protein
625	Smlt2398	hypothetical protein
626	Smlt2399	hypothetical protein
627	Smlt2400	hypothetical protein
628	Smlt2401	hypothetical protein
629	Smlt2403	hypothetical protein
630	Smlt2404	putative transmembrane protein
631	Smlt2405	putative amidase
632	Smlt2406	hypothetical protein
633	Smlt2407	hypothetical protein
634	Smlt2409	putative MerR family transcriptional regulator
635	Smlt2410	putative mercuric transport protein
636	Smlt2411	putative mercuric transport protein periplasmic protein
637	Smlt2412	putative mercuric reductase
638	Smlt2413	putative transposase
639	Smlt2414	putative ISXac3 like transposase for insertion sequence element
640	Smlt2416	putative insertion sequence protein
641	Smlt2417	putative insertion element hypothetical protein
642	Smlt2418	putative monooxygenase
643	Smlt2419	putative transcriptional regulator

644	Smlt2420	putative transmembrane protein
645	Smlt2424	putative arsenate reductase
646	Smlt2427	putative monooxygenase
647	Smlt2428	putative ArsR family regulatory protein
648	Smlt2431	hypothetical protein
649	Smlt2432	putative outer membrane efflux protein
650	Smlt2433	putative cation efflux protein
651	Smlt2434	putative cation efflux system protein
652	Smlt2435	hypothetical protein
653	Smlt2438	hypothetical protein
654	Smlt2439	hypothetical protein
655	Smlt2440	putative copper-transporting p-type ATPase
656	Smlt2441	putative transmembrane transporter protein
657	Smlt2442	putative copper resistance protein
658	Smlt2443	hypothetical protein
659	Smlt2444	hypothetical protein
660	Smlt2445	putative IS3/IS911 family transposase
661	Smlt2446	putative cytochrome c family protein
662	Smlt2447	putative copper resistance protein
663	Smlt2448	putative copper resistance protein
664	Smlt2449	putative transcriptional regulator of copper resistance
665	Smlt2450	putative transcriptional repressor protein
666	Smlt2451	hypothetical protein
667	Smlt2452	hypothetical protein
668	Smlt2453	putative transposon cointegrate resolution protein
669	Smlt2454	putative conjugative transposon DNA recombination protein
670	Smlt2455	hypothetical protein
671	Smlt2460	hypothetical protein
672	Smlt2461	putative transmembrane protein
673	Smlt2462	putative transmembrane permease
674	Smlt2463	putative transcriptional regulator
675	Smlt2464	putative phage integrase
676	Smlt2465	putative transposon Tn5044/Tn3926 transposase
677	Smlt2466	hypothetical protein
678	Smlt2467	hypothetical protein
679	Smlt2468	hypothetical protein
680	Smlt2469	hypothetical protein
681	Smlt2470	hypothetical protein

682	Smlt2474	hypothetical protein
683	Smlt2475	hypothetical protein
684	Smlt2477	putative transmembrane protein
685	Smlt2479	putative ISXac3 like transposase
686	Smlt2479A	hypothetical protein
687	Smlt2481	hypothetical protein
688	Smlt2482	hypothetical protein
689	Smlt2482A	hypothetical protein
690	Smlt2482B	putative phage-like protein
691	Smlt2486	putative phage-like protein
692	Smlt2488	hypothetical protein
693	Smlt2490	putative ISXac3 like transposase family protein
694	Smlt2491	hypothetical protein
695	Smlt2492	hypothetical protein
696	Smlt2493	hypothetical protein
697	Smlt2494	hypothetical protein
698	Smlt2495	hypothetical protein
699	Smlt2496	hypothetical protein
700	Smlt2497	putative transmembrane protein
701	Smlt2498	putative transmembrane efflux protein
702	Smlt2499	hypothetical protein
703	Smlt2500	hypothetical protein
704	Smlt2501	hypothetical protein
705	Smlt2502	hypothetical protein
706	Smlt2503	putative xanthine dehydrogenase YagR, molybdenum binding subunit
707	Smlt2504	putative xanthine dehydrogenase YagS, fad binding subunit
708	Smlt2505	putative xanthine dehydrogenase iron-sulfur-binding subunit
709	Smlt2506	hypothetical protein
710	Smlt2519	hypothetical protein
711	Smlt2521	hypothetical protein
712	Smlt2522	hypothetical protein
713	Smlt2525	hypothetical protein
714	Smlt2526	putative transmembrane protein
715	Smlt2527	putative iron-sulfur binding oxidoreductase
716	Smlt2530	putative DNA ligase family protein
717	Smlt2531	hypothetical protein
718	Smlt2535	putative sensors of blue light using FAD protein
719	Smlt2536	hypothetical protein

720	Smlt2537	putative managnese containing catalase
721	Smlt2538	hypothetical protein
722	Smlt2547	putative transmembrane protein
723	Smlt2549	hypothetical protein
724	Smlt2550	putative transmembrane protein
725	Smlt2551	putative transmembrane protein
726	Smlt2552	putative isochorismatase
727	Smlt2553	hypothetical protein
728	Smlt2554	hypothetical protein
729	Smlt2555	putative LacI family transcriptional regulator
730	Smlt2556	putative multiphosphoryl transfer protein
731	Smlt2557	putative 1-phosphofructokinase
732	Smlt2558	putative PTS system fructose-specific transporter subunit IIBC
733	Smlt2559	putative outer membrane regulator of pathogenicity factors protein
734	Smlt2560	putative oxidoreductase
735	Smlt2563	putative penicillin-binding protein/beta-lactamase
736	Smlt2566	putative TonB dependent receptor protein
737	Smlt2567	putative transmembrane transporter
738	Smlt2571	putative phosphate selective porin
739	Smlt2572	putative ABC transporter transmembrane permease
740	Smlt2573	putative ATP-binding ABC transporter protein
741	Smlt2574	putative ABC transporter protein
742	Smlt2575	hypothetical protein
743	Smlt2578	hypothetical protein
744	Smlt2580	hypothetical protein
745	Smlt2581	hypothetical protein
746	Smlt2582	putative 5,10-methylenetetrahydrofolate reductase
747	Smlt2583	putative LysR family transcriptional regulator
748	Smlt2584	putative NADH-dependent FMN reductase
749	Smlt2585	hypothetical protein
750	Smlt2586	5-methyltetrahydropteroyltriglutamate-- homocysteine methyltransferase
751	Smlt2587	hypothetical protein
752	Smlt2588	putative trypsin protease
753	Smlt2597	putative NAD(P)H-dependent FMN reductase
754	Smlt2598	hypothetical protein
755	Smlt2607	putative transmembrane protein
756	Smlt2608	hypothetical protein
757	Smlt2609	putative X-Pro dipeptidyl-peptidase

758	Smlt2610	hypothetical protein
759	Smlt2611	putative endonuclease
760	Smlt2612	putative haloacid dehalogenase-like hydrolase
761	Smlt2613	putative transmembrane protein
762	Smlt2616	putative LysR family regulatory protein
763	Smlt2617	hypothetical protein
764	Smlt2618	putative Beta propeller repeat-containing protein
765	Smlt2619	hypothetical protein
766	Smlt2620	hypothetical protein
767	Smlt2631	hypothetical protein
768	Smlt2637	putative trehalose synthase
769	Smlt2638	putative dehydrogenase
770	Smlt2639	hypothetical protein
771	Smlt2640	putative dehydrogenase/oxidoreductase
772	Smlt2641	putative transmembrane protein
773	Smlt2642	putative macrolide-specific ABC-type efflux carrier
774	Smlt2643	putative HlyD family secretion protein
775	Smlt2644	putative transmembrane protein
776	Smlt2645	putative two-component regulatory system family, response regulator protein
777	Smlt2646	putative two-component regulatory system family, sensor histidine kinase
778	Smlt2647	putative transmembrane protein
779	Smlt2648	hypothetical protein
780	Smlt2649	hypothetical protein
781	Smlt2650	putative ferric siderophore receptor
782	Smlt2652	conjugal transfer protein TrbP
783	Smlt2659	hypothetical protein
784	Smlt2660	hypothetical protein
785	Smlt2661	putative transmembrane protein
786	Smlt2662	putative transmembrane protein
787	Smlt2663	putative transmembrane protein
788	Smlt2664	putative RNA polymerase sigma factor
789	Smlt2666	putative TonB-dependent ferric siderophore receptor
790	Smlt2668	hypothetical protein
791	Smlt2669	putative transmembrane anchor protein
792	Smlt2670	hypothetical protein
793	Smlt2671	putative molybdopterin oxidoreductase
794	Smlt2672	putative formate dehydrogenase associated protein
795	Smlt2675	putative carboxymuconolactone decarboxylase family protein

796	Smlt2676	putative AraC family transcriptional regulator
797	Smlt2677	putative TetR family regulatory protein
798	Smlt2678	putative transmembrane protein
799	Smlt2679	hypothetical protein
800	Smlt2680	putative lipoprotein
801	Smlt2681	putative lipoprotein
802	Smlt2682	putative ABC transporter transmembrane permease
803	Smlt2683	putative ABC-transporter ATP binding protein
804	Smlt2684	hypothetical protein
805	Smlt2685	putative transmembrane protein
806	Smlt2686	putative LysR family transcriptional regulator
807	Smlt2689	hypothetical protein
808	Smlt2690	hypothetical protein
809	Smlt2691	putative cation resistance transporter protein
810	Smlt2692	putative copper resistance transporter protein
811	Smlt2693	putative two-component response regulator transcriptional regulator
812	Smlt2694	putative two-component sensor histidine kinase
813	Smlt2695	putative LysR family transcriptional regulator
814	Smlt2697	putative cation efflux-related membrane protein
815	Smlt2698	putative cobalt-zinc-cadmium resistance protein
816	Smlt2699	putative outer membrane efflux protein
817	Smlt2700	putative two-component response regulator protein
818	Smlt2701	putative two-component system sensor histidine kinase
819	Smlt2707	hypothetical protein
820	Smlt2710	putative two-component sensor kinase/response regulator fusion protein
821	Smlt2712	hypothetical protein
822	Smlt2713	hypothetical protein
823	Smlt2714	putative TonB dependent protein
824	Smlt2715	putative transmembrane sensor histidine kinase transcriptional regulator
825	Smlt2716	putative ECF sigma factor
826	Smlt2718	putative LysR family regulatory protein
827	Smlt2719	putative pirin domain-containing protein
828	Smlt2720	putative isochorismatase
829	Smlt2721	putative isochorismatase
830	Smlt2723	hypothetical protein
831	Smlt2730	putative general secretory pathway protein
832	Smlt2731	putative general secretion pathway protein H
833	Smlt2732	putative general secretion pathway protein I

834	Smlt2733	putative general secretion pathway protein J
835	Smlt2735	putative transposase
836	Smlt2738	putative alkaline phosphatase I
837	Smlt2740	putative general secretion pathway protein F
838	Smlt2741	putative general secretion pathway protein E
839	Smlt2742	putative general secretion pathway protein D
840	Smlt2743	putative general secretion pathway protein M
841	Smlt2744	putative general secretion pathway protein L
842	Smlt2745	putative general secretion pathway protein K
843	Smlt2746	putative general secretion pathway protein G
844	Smlt2747	hypothetical protein
845	Smlt2748	hypothetical protein
846	Smlt2754	putative transmembrane AzlC amino acid transport protein
847	Smlt2755	putative transmembrane amino acid transporter protein
848	Smlt2756	putative glycogen debranching enzyme
849	Smlt2757	putative alpha amylase/glycosyl hydrolase
850	Smlt2758	putative 4-alpha-glucanotransferase
851	Smlt2759	putative Maltooligosyltrehalose trehalohydrolase protein
852	Smlt2760	glycogen branching enzyme
853	Smlt2761	glycogen synthase
854	Smlt2764	putative molybdenum transport-related, ATP-binding protein
855	Smlt2765	putative ABC molybdenum transport-related membrane protein
856	Smlt2766	putative molybdenum transport-related, substrate-binding protein
857	Smlt2768	putative oxygen-independent coproporphyrinogen III oxidase
858	Smlt2769	putative MFS transmembrane nitrite extrusion transporter protein
859	Smlt2770	putative rotamase/peptidyl-prolyl cis-trans isomerase family protein
860	Smlt2771	putative respiratory nitrate reductase subunit gamma
861	Smlt2772	putative respiratory nitrate reductase subunit delta
862	Smlt2773	putative respiratory nitrate reductase subunit
863	Smlt2774	putative respiratory nitrate reductase subunit alpha
864	Smlt2775	putative major facilitator superfamily transmembrane nitrite extrusion protein
865	Smlt2776	hypothetical protein
866	Smlt2777	putative molybdopterin-guanine dinucleotide biosynthesis protein A
867	Smlt2778	putative molydopterin converting factor subunit 2 protein
868	Smlt2779	putative molybdopterin converting factor subunit 1
869	Smlt2780	putative molybdopterin biosynthesis protein
870	Smlt2781	bifunctional molybdenum cofactor biosynthesis protein MoaC/MogA
871	Smlt2782	putative molybdenum cofactor biosynthesis protein A

872	Smlt2783	hypothetical protein
873	Smlt2784	putative transmembrane protein
874	Smlt2785	hypothetical protein
875	Smlt2786	hypothetical protein
876	Smlt2787	hypothetical protein
877	Smlt2788	putative transmembrane protein
878	Smlt2789	hypothetical protein
879	Smlt2790	putative transmembrane protein
880	Smlt2791	hypothetical protein
881	Smlt2792	putative Rhs family transmembrane protein
882	Smlt2793	hypothetical protein
883	Smlt2794	putative alpha/beta hydrolase fold protein
884	Smlt2795	putative transcriptional regulator
885	Smlt2796	putative transmembrane fusaric acid resistance efflux protein
886	Smlt2797	putative multidrug resistance protein
887	Smlt2798	putative outer membrane multidrug efflux protein
888	Smlt2825	putative luciferase-like monooxygenase
889	Smlt2835	putative TonB-dependent outer membrane protein
890	Smlt2836	hypothetical protein
891	Smlt2844	hypothetical protein
892	Smlt2845	putative TonB dependent receptor protein
893	Smlt2847	putative dioxygenase
894	Smlt2849	putative transmembrane FecR family iron uptake regulator protein
895	Smlt2850	putative TonB dependent extracellular heme-binding protein
896	Smlt2858	putative iron transporter
897	Smlt2859	putative transporter transmembrane protein
898	Smlt2860	hypothetical protein
899	Smlt2863	hypothetical protein
900	Smlt2895	putative ankyrin repeat-containing exported protein
901	Smlt2897	hypothetical protein
902	Smlt2898	cytosine deaminase
903	Smlt2899	hypothetical protein
904	Smlt2902	hypothetical protein
905	Smlt2913	putative AraC family transcriptional regulator
906	Smlt2920	hypothetical protein
907	Smlt2923	putative LysR family transcriptional regulator
908	Smlt2924	putative thioredoxin DsbA family
909	Smlt2925	putative exported cyclase

910	Smlt2934	hypothetical protein
911	Smlt2936	putative FecR iron transport regulator family protein
912	Smlt2938	putative iron regulated lipoprotein
913	Smlt2955	putative acetyltransferase
914	Smlt2957	putative antibiotic monooxygenase biosynthesis protein
915	Smlt2958	putative transmembrane protein
916	Smlt2977	putative transmembrane protein
917	Smlt2984	putative NADPH dependent flavodoxin-like protein
918	Smlt2986	hypothetical protein
919	Smlt2988	hypothetical protein
920	Smlt2989	hypothetical protein
921	Smlt2990	hypothetical protein
922	Smlt2991	hypothetical protein
923	Smlt2992	putative bacteriophage protein
924	Smlt2993	hypothetical protein
925	Smlt2994	hypothetical protein
926	Smlt2995	putative lipoprotein
927	Smlt2996	hypothetical protein
928	Smlt2997	putative type IV secretion system transmembrane protein
929	Smlt2998	hypothetical protein
930	Smlt2999	putative type IV secretion conjugal transfer protein
931	Smlt3000	putative type IV secretion system transmembrane protein
932	Smlt3001	putative type IV secretion system protein
933	Smlt3002	putative type IV secretion system conjugal protein
934	Smlt3003	putative type IV secretion system conjugal transfer protein
935	Smlt3004	secretion system protein
936	Smlt3005	putative VirB9
937	Smlt3006	putative putative conjugative transfer protein
938	Smlt3007	hypothetical protein
939	Smlt3008	putative transmembrane conjugative DNA transfer protein
940	Smlt3009	hypothetical protein
941	Smlt3012	putative MerR family transcriptional regulator
942	Smlt3013	putative NADH oxidoreductase
943	Smlt3014	putative transmembrane protein
944	Smlt3015	putative transmembrane protein
945	Smlt3016	putative transmembrane protein
946	Smlt3017	hypothetical protein
947	Smlt3018	hypothetical protein

948	Smlt3020	putative FAD binding monooxygenase
949	Smlt3021	putative TetR family transcriptional regulator
950	Smlt3023	hypothetical protein
951	Smlt3024	hypothetical protein
952	Smlt3025	hypothetical protein
953	Smlt3027	putative alcohol dehydrogenase
954	Smlt3028	hypothetical protein
955	Smlt3029	hypothetical protein
956	Smlt3031	putative phage-like protein
957	Smlt3033	putative ankyrin repeat-containing protein
958	Smlt3034	hypothetical protein
959	Smlt3035	hypothetical protein
960	Smlt3036	hypothetical protein
961	Smlt3037	hypothetical protein
962	Smlt3038	hypothetical protein
963	Smlt3039	putative UV protection and mutation protein
964	Smlt3040	hypothetical protein
965	Smlt3041	hypothetical protein
966	Smlt3042	putative ISXac3 like transposase
967	Smlt3045	hypothetical protein
968	Smlt3048	putative Hep Hag family adhesin
969	Smlt3049	hypothetical protein
970	Smlt3050	hypothetical protein
971	Smlt3051	putative conjugal transfer protein TraA
972	Smlt3052	hypothetical protein
973	Smlt3053	putative type IV secretory protein conjugation protein TraD
974	Smlt3054	putative ankyrin repeat-containing protein
975	Smlt3055	hypothetical protein
976	Smlt3056	putative transmembrane protein
977	Smlt3057	hypothetical protein
978	Smlt3058	hypothetical protein
979	Smlt3059	putative transmembrane protein
980	Smlt3060	putative transmembrane protein
981	Smlt3061	hypothetical protein
982	Smlt3062	hypothetical protein
983	Smlt3062AA	hypothetical protein
984	Smlt3062A	hypothetical protein
985	Smlt3065	hypothetical protein

986	Smlt3066	hypothetical protein
987	Smlt3067	hypothetical protein
988	Smlt3068	hypothetical protein
989	Smlt3069	putative plasmid partitioning like protein
990	Smlt3070	hypothetical protein
991	Smlt3071	putative type IV pilus protein
992	Smlt3073	hypothetical protein
993	Smlt3074	hypothetical protein
994	Smlt3075	hypothetical protein
995	Smlt3076	hypothetical protein
996	Smlt3077	hypothetical protein
997	Smlt3078	hypothetical protein
998	Smlt3079	hypothetical protein
999	Smlt3080	hypothetical protein
1000	Smlt3081	putative ABC transporter permease
1001	Smlt3082	hypothetical protein
1002	Smlt3084	hypothetical protein
1003	Smlt3085	hypothetical protein
1004	Smlt3086	putative phage-related integrase
1005	Smlt3095	hypothetical protein
1006	Smlt3104	putative phage-like protein
1007	Smlt3111	hypothetical protein
1008	Smlt3112	hypothetical protein
1009	Smlt3114	putative beta-lactamase
1010	Smlt3116	putative transmemrbane protein
1011	Smlt3117	hypothetical protein
1012	Smlt3118	putative TonB receptor like protein
1013	Smlt3120	hypothetical protein
1014	Smlt3124	hypothetical protein
1015	Smlt3129	hypothetical protein
1016	Smlt3134	putative LysR family transcriptional regulator
1017	Smlt3135	putative oxidoreductase
1018	Smlt3146	hypothetical protein
1019	Smlt3147	hypothetical protein
1020	Smlt3173	hypothetical protein
1021	Smlt3200	hypothetical protein
1022	Smlt3202	putative exported alkaline phosphatase D
1023	Smlt3203	putative TonB-dependent receptor

1024	Smlt3230	hypothetical protein
1025	Smlt3262	putative AraC family transcriptional regulator
1026	Smlt3263	putative transmembrane major facilitator superfamily transport protein
1027	Smlt3264	hypothetical protein
1028	Smlt3303	putative transmembrane protein
1029	Smlt3309	hypothetical protein
1030	Smlt3335	putative penicillin binding protein
1031	Smlt3344	hypothetical protein
1032	Smlt3345	short chain dehydrogenase
1033	Smlt3346	putative O6-methylguanine-DNA methyltransferase
1034	Smlt3359	hypothetical protein
1035	Smlt3360	hypothetical protein
1036	Smlt3367	hypothetical protein
1037	Smlt3413	putative UDP-glucose 4-epimerase
1038	Smlt3422	hypothetical protein
1039	Smlt3457	putative D-amino acid dehydrogenase small subunit
1040	Smlt3526	putative outer membrane lipoprotein
1041	Smlt3527	putative outer membrane lipoprotein
1042	Smlt3546	hypothetical protein
1043	Smlt3566	putative glyoxalase/belomycin resistance protein
1044	Smlt3580	putative 3-demethylubiquinone-9 3-methyltransferase
1045	Smlt3585	hypothetical protein
1046	Smlt3586	hypothetical protein
1047	Smlt3587	putative transmembrane protein
1048	Smlt3591	putative transmembrane protein
1049	Smlt3593	hypothetical protein
1050	Smlt3606	putative plasmid stabilisation system protein
1051	Smlt3607	hypothetical protein
1052	Smlt3612	hypothetical protein
1053	Smlt3614	hypothetical protein
1054	Smlt3615	putative aminoglycoside 6'-N-acetyltransferase
1055	Smlt3645	putative TonB dependent receptor protein
1056	Smlt3653	putative short chain dehydrogenase
1057	Smlt3654	putative LysR family transcriptional regulator
1058	Smlt3655	putative histone protein
1059	Smlt3656	putative ABC transporter protein
1060	Smlt3683	putative O-antigen-like protein
1061	Smlt3694	putative transmembrane protein

1062	Smlt3697	putative ferric-enterobactin hydrolase
1063	Smlt3698	hypothetical protein
1064	Smlt3699	hypothetical protein
1065	Smlt3721	putative transmembrane Na+/H+ antiporter
1066	Smlt3729	hypothetical protein
1067	Smlt3730	putative transmembrane two component system sensor histidine kinase
1068	Smlt3731	putative MltA scaffolding protein
1069	Smlt3749	putative exported lipase
1070	Smlt3757	putative prepilin peptidase dependent protein D
1071	Smlt3763	hypothetical protein
1072	Smlt3764	hypothetical protein
1073	Smlt3768	hypothetical protein
1074	Smlt3770	putative thioesterase family protein
1075	Smlt3772	hypothetical protein
1076	Smlt3778	hypothetical protein
1077	Smlt3796	hypothetical protein
1078	Smlt3828	putative transmembrane protein
1079	Smlt3843a	putative formate dehydrogenase-o, major subunit
1080	Smlt3845	putative formate dehydrogenase, iron-sulfur subunit
1081	Smlt3846	putative formate dehydrogenase, cytochrome b556 (FDN) subunit
1082	Smlt3848	putative formate dehydrogenase FdhE
1083	Smlt3849	putative selenocysteinyl-tRNA(SeC) synthase
1084	Smlt3850	putative selenocysteine-specific elongation factor
1085	Smlt3851	putative selenide, water dikinase
1086	Smlt3852	putative helix-turn-helix DNA binding protein
1087	Smlt3853	hypothetical protein
1088	Smlt3862	putative transposon-like protein
1089	Smlt3888	hypothetical protein
1090	Smlt3904	putative transmembrane protein
1091	Smlt3911	hypothetical protein
1092	Smlt3922	putative short-chain dehydrogenase
1093	Smlt3929	putative cationic amino acid transporter
1094	Smlt3938	putative transposase
1095	Smlt3953	hypothetical protein
1096	Smlt3983	putative glyoxlase/bleomycin resistance protein
1097	Smlt3984	putative hydrolase
1098	Smlt3985	putative TetR family transcriptional regulator
1099	Smlt3990	hypothetical protein

1100	Smlt3999A	hypothetical protein
1101	Smlt4005	hypothetical protein
1102	Smlt4006	hypothetical protein
1103	Smlt4009	hypothetical protein
1104	Smlt4010	hypothetical protein
1105	Smlt4011	hypothetical protein
1106	Smlt4012	hypothetical protein
1107	Smlt4016	hypothetical protein
1108	Smlt4017	hypothetical protein
1109	Smlt4074	putative GFO/IDH/MocA family oxidoreductase
1110	Smlt4107	putative acetyltransferase
1111	Smlt4140	hypothetical protein
1112	Smlt4142	putative TetR family transcriptional regulator
1113	Smlt4144	hypothetical protein
1114	Smlt4146	hypothetical protein
1115	Smlt4147	hypothetical protein
1116	Smlt4148	putative RHS family protein
1117	Smlt4150	hypothetical protein
1118	Smlt4152	hypothetical protein
1119	Smlt4153	putative transmembrane protein
1120	Smlt4154	putative AraC family regulatory protein
1121	Smlt4155	putative glutatione S-transferase
1122	Smlt4156	putative transmembrane protein
1123	Smlt4157	putative DNA-binding protein HNS
1124	Smlt4158	putative transmembrane DoxX family protein
1125	Smlt4159	putative beta-lactamase
1126	Smlt4160	hypothetical protein
1127	Smlt4161	putative glycosyltransferase family protein
1128	Smlt4162	putative phage regulatory protein
1129	Smlt4163	hypothetical protein
1130	Smlt4164	hypothetical protein
1131	Smlt4189	hypothetical protein
1132	Smlt4204	hypothetical protein
1133	Smlt4208	putative transmembane sensor histidine kinase transcriptional regulator
1134	Smlt4209	putative two component response regulator
1135	Smlt4210	hypothetical protein
1136	Smlt4211	putative beta-lactamase
1137	Smlt4213	hypothetical protein

1138	Smlt4220	putative PEPSY domain transmembrane protein
1139	Smlt4221	putative flavodoxin NAD-binding oxidoreductase
1140	Smlt4223	hypothetical protein
1141	Smlt4228	hypothetical protein
1142	Smlt4231	hypothetical protein
1143	Smlt4232	putative alkaline phosphatase 4
1144	Smlt4233	putative family S11 unassigned peptidase
1145	Smlt4236	hypothetical protein
1146	Smlt4238	hypothetical protein
1147	Smlt4255	putative DNA-binding protein
1148	Smlt4256	hypothetical protein
1149	Smlt4283	putative short-chain dehydrogenase/oxidoreductase
1150	Smlt4285	putative transmembrane protein
1151	Smlt4304	putative chaperone protein
1152	Smlt4331	hypothetical protein
1153	Smlt4332	hypothetical protein
1154	Smlt4342	ehpr protein
1155	Smlt4343	putative deoR family transcriptional regulator
1156	Smlt4362	hypothetical protein
1157	Smlt4369	putative transposase
1158	Smlt4372	putative ADP-ribosylating transferase
1159	Smlt4373	hypothetical protein
1160	Smlt4374	hypothetical protein
1161	Smlt4375	hypothetical protein
1162	Smlt4376	hypothetical protein
1163	Smlt4378	putative transmembrane protein
1164	Smlt4380	hypothetical protein
1165	Smlt4381	putative cold shock protein
1166	Smlt4382	hypothetical protein
1167	Smlt4383	hypothetical protein
1168	Smlt4384	putative carbon-nitrogen hydrolase
1169	Smlt4386	hypothetical protein
1170	Smlt4387	putative TonB dependent receptor protein
1171	Smlt4389	hypothetical protein
1172	Smlt4391	putative exopolysaccharide synthesis protein
1173	Smlt4392A	hypothetical protein
1174	Smlt4394	hypothetical protein
1175	Smlt4395	putative serine protease

1176	Smlt4396	hypothetical protein
1177	Smlt4398	putative cytochrome O ubiquinol oxidase subunit IV
1178	Smlt4402	putative GntR family transcriptional regulator
1179	Smlt4403	hypothetical protein
1180	Smlt4404	hypothetical protein
1181	Smlt4410	putative TonB dependent ferric enterobactin receptor
1182	Smlt4411	putative transmembrane protein
1183	Smlt4416	putative oligopeptidase
1184	Smlt4417	hypothetical protein
1185	Smlt4418	hypothetical protein
1186	Smlt4423	putative AfaD non-fimbrial adhesin
1187	Smlt4424	putative transmembrane protein
1188	Smlt4426	hypothetical protein
1189	Smlt4428	hypothetical protein
1190	Smlt4429	putative transmembrane protein
1191	Smlt4430	putative Rhs family exported protein
1192	Smlt4444A	hypothetical protein
1193	Smlt4447B	hypothetical protein
1194	Smlt4452	putative cell surface haemagluttinin protein
1195	Smlt4453	putative two-partner secretion system protein
1196	Smlt4455	putative major royal jelly-like exported protein
1197	Smlt4472	hypothetical protein
1198	Smlt4474	putative multidrug efflux system outer membrane protein
1199	Smlt4475	putative multidrug efflux protein
1200	Smlt4476	putative drug resistance efflux protein
1201	Smlt4477	putative two-component system sensor histidine kinase
1202	Smlt4478	putative two-component system response regulator
1203	Smlt4487	hypothetical protein
1204	Smlt4509	hypothetical protein
1205	Smlt4515	putative cellulose/chitin-binding protein
1206	Smlt4516	putative secreted cellulose/chitin-binding protein
1207	Smlt4520	hypothetical protein
1208	Smlt4527	putative transmembrane protein
1209	Smlt4532	hypothetical protein
1210	Smlt4540	putative two-component sensor histidine kinase
1211	Smlt4541	hypothetical protein
1212	Smlt4543	putative exported lipoprotein
1213	Smlt4546	putative transmembrane protein

1214	Smlt4548	putative phage-like protein
1215	Smlt4576	putative ABC transporter permease
1216	Smlt4577	putative ATP-binding ABC transporter protein
1217	Smlt4578	putative transmembrane protein
1218	Smlt4583	putative transmembrane protein
1219	Smlt4584	hypothetical protein
1220	Smlt4593	hypothetical protein
1221	Smlt4602	putative exonuclease
1222	Smlt4605	hypothetical protein
1223	Smlt4606	putative efflux transporter protein
1224	Smlt4607	putative HlyD family secretion protein
1225	Smlt4608	putative transmembrane efflux pump protein
1226	Smlt4618	hypothetical protein
1227	Smlt4629	putative chaperone heat shock Hsp70 protein
1228	Smlt4630	putative transmembrane heat shock chaperone
1229	Smlt4631	putative transmembrane chaperone heat shock protein
1230	Smlt4666	hypothetical protein
